# Plants against *Helicobacter pylori* to combat resistance: An ethnopharmacological review

**DOI:** 10.1016/j.btre.2020.e00470

**Published:** 2020-05-21

**Authors:** Doha Abou Baker

**Affiliations:** Medicinal and Aromatic Plants Dept., Pharmaceutical and Drug Industries Division, National Research Centre, Cairo, Egypt

**Keywords:** *Helicobacter pylori*, Medicinal plants, Secondary metabolites, Combat antibiotic resistance

## Abstract

Worldwide, *Helicobacter pylori* (*H. pylori*) is regarded as the major etiological agent of peptic ulcer and gastric carcinoma. Claiming about 50 percent of the world population is infected with *H. pylori* while therapies for its eradication have failed because of many reasons including the acquired resistance against its antibiotics. Hence, the need to find new anti-*H.pylori* medications has become a hotspot with the urge of searching for alternative, more potent and safer inhibitors. In the recent drug technology scenario, medicinal plants are suggested as repositories for novel synthetic substances. Hitherto, is considered as ecofriendly, simple, more secure, easy, quick, and less toxic traditional treatment technique. This review is to highlight the anti-*H. pylori* medicinal plants, secondary metabolites and their mode of action with the aim of documenting such plants before they are effected by cultures and traditions that is expected as necessity.

## Highlights

• The effectiveness of the eradication therapy of *H. pylori* is hampered by increasing resistance against antibiotics.
• In the recent drug technology scenario, Medicinal plants are repositories for novel synthetic substances.
• Medicinal plants is the ideal therapy to combat resistance.

## Graphical abstract

**Figure:**
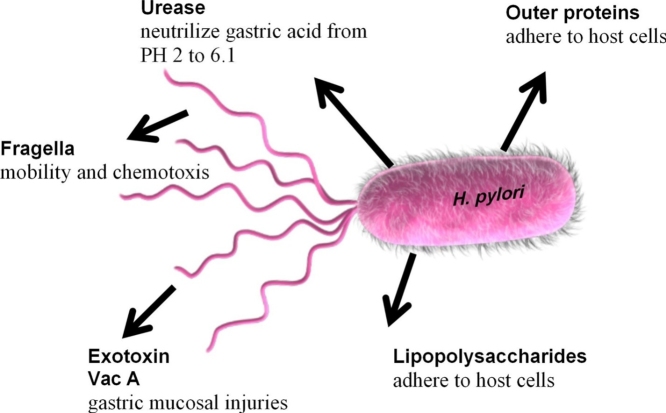

## 1 Introduction

**Fig. 1:**
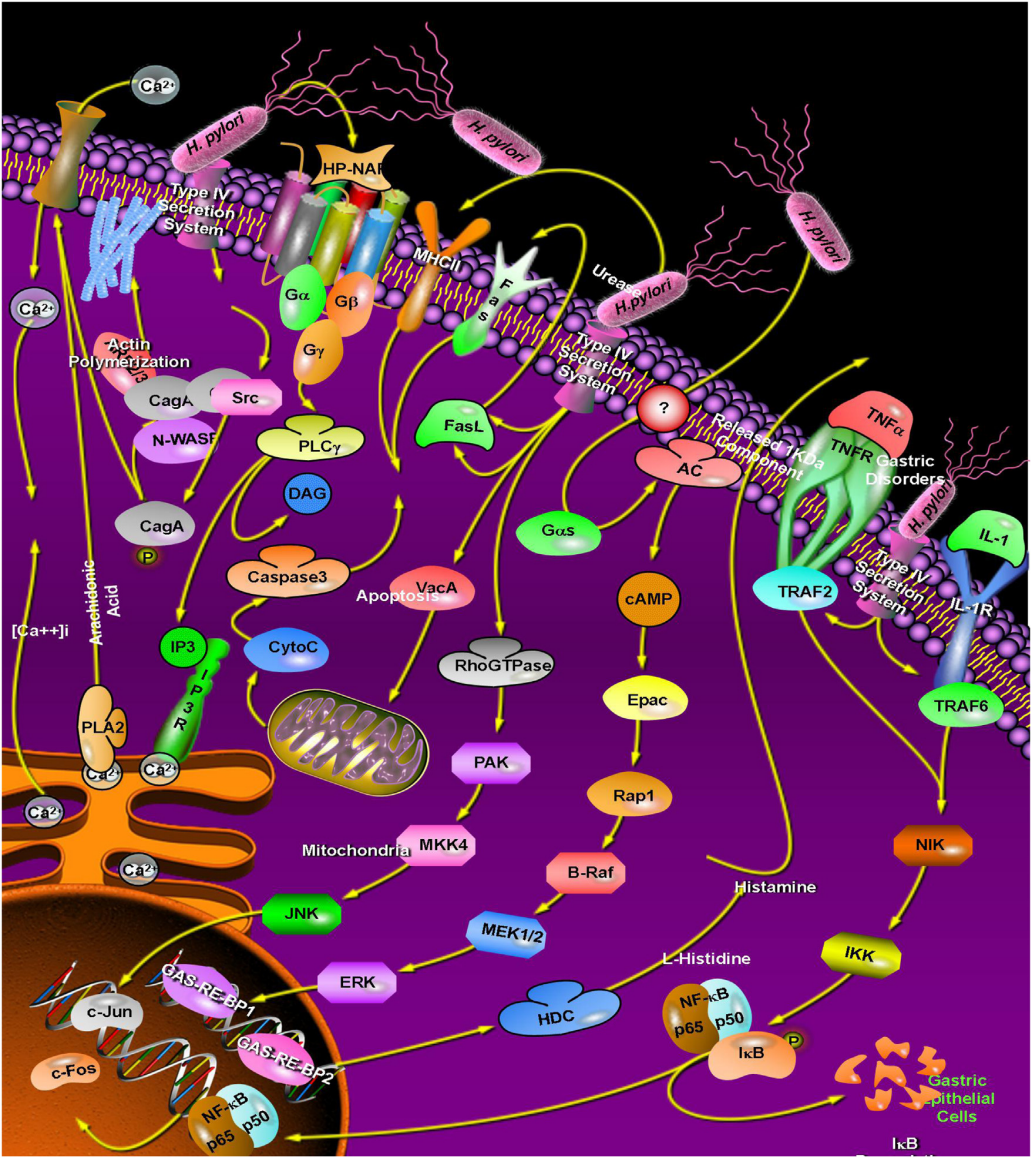
Virulence agents of *H. pylori*. IL: Interleukin; TLR4: Toll-like receptor 4; NF-κB: Nuclear factor-kappaB; NIK: NF-κB-inducing kinase; VacA: Vacuolating cytotoxin A; CagA: Cytotoxin-associated gene antigen; PAK1: p21-activated kinase; IKKα/β: IκB kinase α/β; MAPK: Mitogen-activated protein kinase; MEK1/2: MAPK/ERK kinase 1/2; INF-γ: Interferon-γ; NOD1: Nucleotide-binding oligomerisation domain protein 1; ICAM-1: Intercellular adhesion molecule-1; iNOS: Inducible nitric oxide synthase, COX-2: Cyclooxygenase-2; MKK4: MAPK kinase 4; LPS: Lipopolysaccharide; TNF-α: Tumor necrosis factor-α.

*Helicobacter pylori* (*H. pylori*) is a spiral-shaped Gram-negative bacteria colonized in the gastrointestinal tract. *H. pylori* infection leads to peptic ulceration, gastritis, and gastric carcinoma [1]. About 50 % of the world population is estimated to be infected by this bacterium [2]. The colonization of *H. pylori* is caused by its infectious agents as shown in Fig. 1 and Table 1.

**Table 1:** Virulence agents of *H. pylori*.

Vrulence agent	*H. pylori* Function
Vacuolating cytotoxin A (VacA)	Induce Cyto C release
	Cytotoxicity
Cag Pathogenicity Island (CagPAI)	Induce inflammation
Cag genes (Cag E,G,I,H, L and M)	Coding for 40-kb is a major virulence factor of *H. pylori*.
Urease	Causing epithelium cells toxicity
	Disrupting cell tight junctions
	Buffers stomach acid
	Sheathing antigen
Duodenal ulcer promoting A (DupA)	Induce inflammation
Outer inflammatory protein A (OipA)	Induce inflammation for IL-8
*H. pylori* neutrophil activation protein (HP-NAP)	Activation of neutrophil
BabA	Adhesin
Flagella	Movements through mucin

## 2 Pharmacological therapies

Numerous pharmacological studies have been reported for the eradication of *H. pylori*. Proton-pump inhibitors, antibiotics, bismuth saltsand H2-blockers (intragastric pH control drug) are recommended standard therapies [3]. A few issues may arise upon those eradication therapies, for example, the cost, the high global prevalence and the uprising resistance to available antibiotics. Consequently, some patients undergoing many of these drug regimens experience therapeutic failure [3]. Moreover, these therapies include getting too many medications which might cause side effects that, along with significant cost regarding the treatment, promote inadequate patient compliance. It is extremely desirable to explore for alternative strategies with agents to prevent or manage *H. pylori*-associated gastric tumor.

The quest regarding new anti-*H. pylori* therapies has driven exploration in the field of therapeutic plants. Many studies have been performed on a great number of plant varieties. Natural products exhibit their own anti-*H. pylori* actions via different mechanisms. While therapeutic agents have either antisecretory or healing effects, prophylactic compounds produce their effect via their antioxidant and anti-inflammatory mechanisms.

## 3 Mechanisms of medicinal plants as anti-*H. pylori*

Many natural products have anti-*H. pylori* potentials. The mechanisms of such potentials include urease inhibition, DNA damage, protein synthesis inhibition, and anti-inflammatory effects. In addition to the anti-*H. pylori* effects due to some enzymes like dihydrofolate reductase and myeloperoxidase *N*-acetyltransferase.

### 3.1 Urease inhibition

The potent effect of resveratrol as anti-*H. pylori* is mainly owing to ureaseinhibition [4]. The anti- *H. pylori* actions of *Paeonia lactiflora* roots is due to the hydrophobicity of 1,2,3,4,6-penta-*O*-galloyl-β-d-glucopyranose which facilitates thebinding to membranes leading to the loss of membrane integrity as well as urease inhibition [5]. Both the CHCl_3_ fraction and EtOH extract of *Calophyllum brasiliense* stem bark has been reported to decrease *H. pylori* and urease activity in Wistar rats as confirmed by histopathology [6]. The mode of action of mixed cranberry and oregano water extract may be due to inhibition of proline dehydrogenase and urease activvity [7]. Both*Calotropis procera* and *Acacia nilotica* extracts inhibit urease activity through competitive mechanisms [8].

### 3.2 Oxidative stress

2-Methoxy-1,4-naphthoquinoneexhibits strong anti *H. pylori* action. 2-methoxy-1,4-naphthoquinone is metabolized in *H. pylori* membrane by flavoenzymes and produces a high amount of free radicals that may damage cellular macromolecules and may lead to *H. pylori* death [9].

### 3.3 Anti-adhesion activity

Borage, parsley, and turmeric water extracts are found to be able to decrease adhesion of *H. pylori* [10]. The Liquoriceroot aqueous extract and polysaccharides exhibite strong anti-adhesive activity of human gastric mucosa aliquots with fluorescent-labeled *H. pylori* [11]. The *Pelargonium sidoides* root extract display antiadhesive activity [12]. The diterpene Plaunotol, isolated from the plau-noi leaves, is also found to inhibit adhesion of *H. pylori* as well as inhibition of IL-8 secretion [13].

## 4 Structure activity relationship

**Fig. 2:**
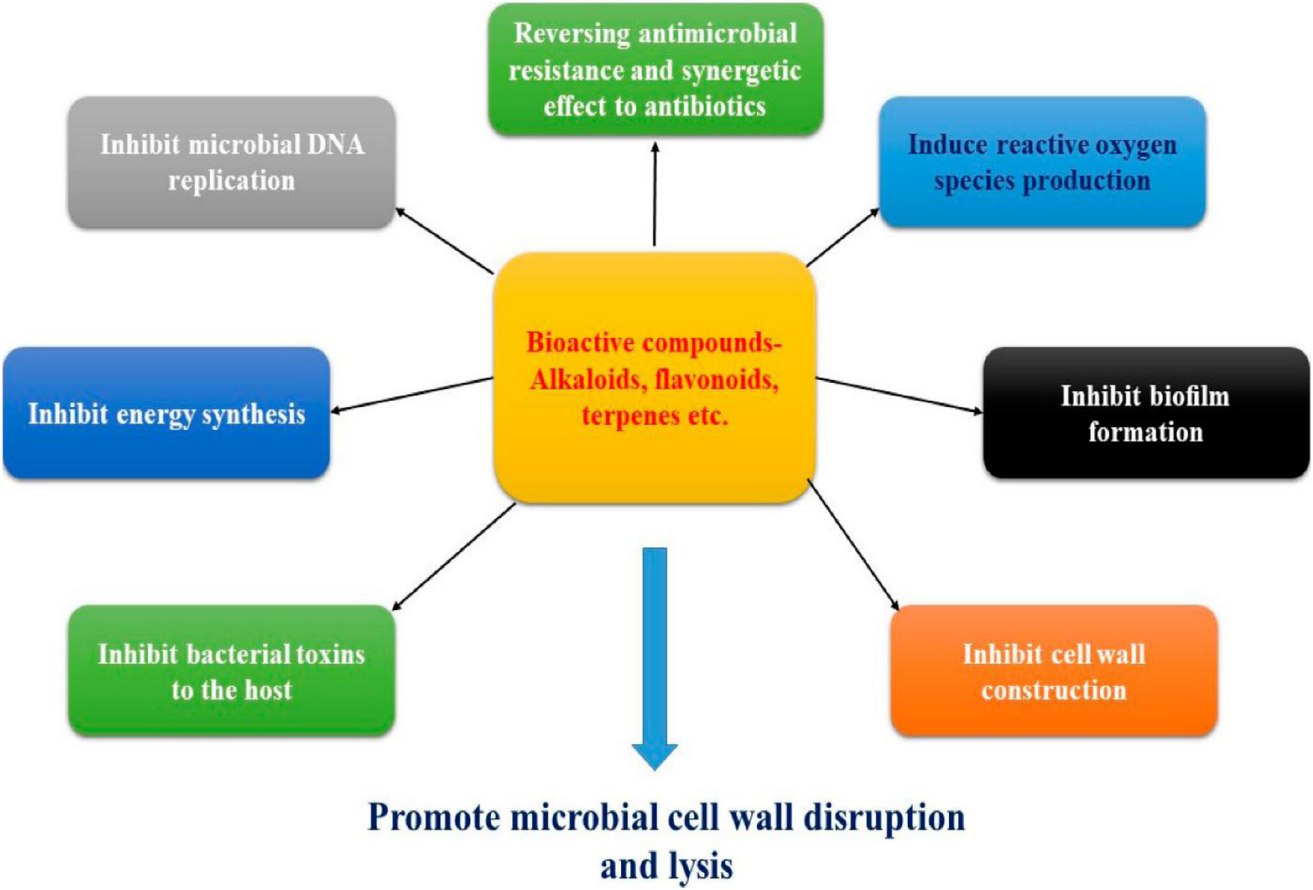
Mechanisms of action of phytocompounds against microorganisms.

Plantswith anti H. pylori activityconsist of various phytocompounds, such as alkaloids, flavonoids, saponins, terpenes, and polysaccharides, which responsible for antimicrobial activity (Fig. 2) are discussed within this review in Table 2.

**Table 2:** Restorative herbs having anti-*H. pylori* action.

Plant Names	Part and extract	Active ingredients responsible for the activity	Activity	Refs.
*Aesculus hippocastanum*	EtOH extract	Saponin (Aescine)	Antisecretory effect	[31]
*Acacia nilotica*	flower aceton extract	Not identified	Urease inhibitor	[8]
*Achillea millefolium*	MeOH extract of aerial parts	Not identified	Antioxidant	[45,46]
*Ageratina pichinchensis*	EtOH extract	3,5-diprenyl-4-hydroxyacetophenone	Maintaenence NO, PG, SH release	[47]
*Ageratum conyzoides*	MeOH extract of the entire plant	Not identified	Not detected	[48]
*Agrimonia pilosa Ledeb.*	Aqueous extract of whole plant	Not identified	Not detected	[49]
*Alchornea triplinervia*	MeOH and EtOAc extracts	Not identified	Antisecretory	[50,51]
			Increase PGE2	
			Decrease gastric injuries	
			Increase mucus	
			Promote epithelial cell	
*Allium sativum*	Oil and aqeous extract	Thiosulfinates	Interfere with cell wall	[52,53,54,55,56]
		Diallyl disulfide	Causing cell lysis and Triggering autolysis	
*Aloe vera*	Polysachharide fraction	Lectins	Increase mucus	[57]
			Inhibit aminopyrin uptake	
			Reduce TNF-α	
*Alpinia speciosa*	EtOH extract of root	Not identified	Inhibit H.pylori	[58]
*Amphipterygium adstringens.*	CH_2_Cl_2_ extract	3a-hydroxymasticadienonic acid, b-sitosterol	Gastroprotective	[59]
		3-*epi*-oleanolic acid		
*Angelica sinensis*	EtOH extract	Polysaccharide indomethacin	Inhibition of MPO activity	[60]
*Anisomeles indica*	Stem and leaves EtOH extract	Not identified	Inhibit IL-12 and TNF-α,	[58]
*Annona cherimola*	Stem and leaves MeOH extract	Not identified	Not detected	[61]
*Anthemis altissima*	Isolated compounds from arial part	Sesquiterpene lactones	Not detected	[62]
		Tatridin-A, sivasinolide, 1-epi-tatridin B, altissin, desacetyl-β-cyclopyrethrosin,		
*Aralia elata*	Root bark	Araloside A	Gastric lesion inhibitor ulcer formation inhibitor	[33]
*Arrabidaea chica*	HydroEtOHic extract of leaves	Flavones and flavonols	Inhibit H. pylori	[63]
*Artemisia ludoviciana*	Leaves and stem aqueous extract	Artemisin	Bactericidal kinetics	[61]
			Morphological degeneration	
*Atractylodes ovata*	EtOH extract	Sesquiterpenoid	-Inhibition of MMP-2	[64]
		Atractylenolide III	-MMP-9 expression	
*Bixa orellana*	EtOH extract of seeds	Not identified	Not detected	[65]
*Boesenbergia rotunda*	EtOH extract	Flavanone	Antioxidant	[66]
		Pinostrobin	Decrease gastric motility	
*Bombax malabaricum*	EtOH extract of root	Not identified	Not detected	[58]
*Boronia pinnata*	Whole shrub extract	Cinnamic acid derivative (boropinic acid)	Anti-ulcer agent	[67]
*Brassica oleracea*	Broccoli sprouts	Not identified	On human volunteers	[68]
*Brazilian propolis*	Propolis extract	3-hydroxy-2,2dimethyl-8-prenylchromane-propenoic acid	Anti-H.pylori invitro	[69]
*Bridelia micrantha*	Acetone and EtOAc extracts of stem bark	Not identified	Anti-inflammatory	[70,71]
*Byrsonima crassa*	Leaves MeOH and CHCl_3_ extracts	Not identified	Immunostimulatory	[72]
*Byrsonima fagifolia*	Leaves MeOH extract	Not identified	Gastroprotective	[73]
			Antidiarrheal	
			Antibacterial Immunomodulatory	
*Byrsonima intermedia*	Leaves MeOH extract	Not identified	Antioxidant	[74]
*Calophyllum b8rasiliense*	Hexane, HydroEtOH extract and Ch_2_Cl_2_ fraction of stem bark	Mixture of chromanone	Decreased urease,	[6,75]
			Reduce H. pylori in pathological analysis	
*Calotropis procera*	Acetone and MeOH extracts of leaves and flowers	Not identified	Urease inhibitor	[8]
*Camellia sinensis*	MeOH and water extracts of young shoots	Catechin	Urease inhibitor	[27,76,77]
			Anti-inflammatory	
*Carum carvi L.*	Fruit MeOH	Not identified	Not detected	[78]
*Casearia sylvestris*	Leaves EtOH extract	Terpenoids	Decrease ulcerative size	[79]
			Eradicate *H. pylori*	
*Chamomilla recutita*	Oil extract of flowers	Catechin	Urease inhibitor	[65,80,81,82]
	70 % aqueous		Decreasegastric mucosal injury	
	MeOH 96 % ethanol			
*Cinnamomum cassia*	Bark aqueous EtOH	Not identified	Suppression of IL-8	[46]
*Cinnamomum verum*	Essential oils of dry bark	Cinnamaldehyde	Urease inhibitor	[83,84,85,86]
*Cistus laurifolius*	Flowers CHCl_3_ fraction	Isorhamnetin	Inhibit ulcer	[87,88]
		Kaempferol 3,7-dimethyl ether, quercetin 3,7-dimethyl ether	Eradicate *H.pylori*	
*Citrus aurantium*	EtOH extract	Monoterpene	indomethacin, ischemia reperfusion	[89]
		b-Myrcene		
*Citrus lemon*	Essential oil	Monoterpene	Mucus production	[90]
		Indomethacin	HSP-70 activation	
		Limonene	Vasoactive intestinal peptide and NO release	
			Maintenance of PGE2 and glutathione levels	
*Cocculus hirsutus*	EtOH extract of leaves	Alkaloids	Anti *H. pylori*	[91]
*Cochlospermum tinctorium*	Acidified EtOH	Polysaccharide	Antioxidant	[40]
		Arabinogalactans II	Immunomodulatory	
*Combretum molle*	Stem bark acetone extract was the best	Flavonoids	Gastroprotective	[92]
*Coptis chinensis*	Rhizome aqueous extract	Alkaloid	Inhibit ulcer	[93]
			Eradicate *H.pylori*	
*Croton reflexifolius*	EtOH extract	Diterpenoid	Gastroprotective	[94]
		Polyalthic acid	Block sulfhydryl groups	
			Inhibit NO synthase	
*Croton sublyratus*	Leaves extract	Terpenoid (Plaunotol)	Suppress IL-8 secretion	[95]
*Cuminum cyminum*	EtOH extracts of seeds	Phenolic compounds	Antioxidant	[96]
*Cuphea aequipetala*	Leaves aqueous extract	Phenolic compounds	Reduce gastric lesions	[61]
			Inhibit ulcer	
*Curcuma amada*	Rhizome 70 % EtOH	Curcumin	Inhibit proton potassium ATPase	[97]
*Cupressus sempervirens*	Essential oil	Monoterpenes	Not detected	[98]
*Curcuma longa*	Polyphenolic rich extract of the root	Curcumin	Chemo-preventative	[99]
*Cymbopogon citratus*	Essential oil	Terpenes	Inhibit COX	[98]
			Inhibit NO synthase Activate K^+^ATP channel and α2 receptors.	
*Cyrtocarpa procera*	Hexane extracts from stem bark	Not identified	Gastroprotective	[59,61,100]
			Anti-inflammatory	
*Davilla elliptica*	Leaves MeOH extract	Not identified	Anti-inflammatory Gastroprotective	[101]
*Davilla nítida*	Leaves MeOH extract	Not identified	Anti-inflammatory Gastroprotective	[101]
*Daucus carota*	Essential oil of seed	Carvacrol and nerol	Decrease pH	[102]
*Derris trifoliate*	Petroleum ether and stemCHCl_3_ extracts	Not identified	Eradicate *H. Pylori*	[103]
			Gastroprotective	
*Desmostachya bipinnata*	Wholeplant	Flavonoids (4-methoxy quercetin-7-O-glucoside)	Chemopreventive agent	[104,105]
	Diethyl ether extract			
*Dittrichia viscosa*	Aerial parts essential oil (Oxygenated fractions)	3-methoxy cuminyl isobutyrate	Antibacterial action	[81,106]
*Eucalyptus torelliana*	Hexane extract of leaves	Saponin and taninns	Decrease gastric acid	[107]
			Increase pH gastric juice	
*Eugenia caryophillus*	EtOH extracts of flowers	Eugenol	Increase activity at acidic pH	[84,108]
*Eugenia caryophyllata*	Flowers aqueous extract	Essential oil	Anti-inflammatory	[49]
*Eupatorium aschenbornianum*	EtOH extract	Chromene	Antioxidant activity	[109]
		Encecanescin		
*Evodia rutaecarpa*	Alkaloids rich extract	1-Methyl-2-[(Z)-7-tridecenyl]-4-(1 H)-quinolone	Anti-inflammatory	[110]
			Very strong Anti-H.pylori	
*Feijoa sellowiana*	Fruit Acetone Extract	Flavone	Inhibit H^+^/K^+^ATPase activity and Increase PGE_2_	[111]
*Ferulago campestris*	Root extract	Coumarins (Aegelinol and Benzoyl aegelinol)	Not detected	[112,113,114,115]
*Foeniculum vulgare*	MeOH extract of the seeds	Not identified	Antioxidant	[45,46]
*Garcinia achachairu*	Acidified ethanol of the seeds	Polyisoprenylated benzophenone	Gastroprotective	[116]
		Guttiferone A		
*Geranium wilfordii*	EtOH extracts and EtOAc fraction	1,2,3,6-tetra-O-galloyl-β-d-glucose and corilagin	Not detected	[117]
*Geum iranicum*	Aqueous fraction of the roots	Tannins	Gastroprotective	[118]
		Eugenol		
*Glycyrrhiza glabra*	Water extract of the root	Polysaccharide	Anti-adhesive activity	[11,29]
		Flavonoids (glabridin)	Inhibit dihydrofolate reductase	
			Inhibit DNA gyrase	
*Glycyrrhiza uralensis*	MeOH extract of roots	licoricidin licoisoflavone B	Chemopreventive agents	[119,120]
		licoric		
*Guaiacum coulteri*	Bark MeOH extract	Not identified	Antibacterial action	[61]
*Hancornia speciose*	Hydroalcoholic extract of the bark	Not identified	Antibacterial action	[121]
*Hericium erinaceus*	Hydroalcoholic extract of bark	Not identified	Antibacterial action	[122]
*Hydrastis canadensis*	MeOH extract of rhizome	Isoquinoline alkaloids	Inhibit bacterial efflux pumps, Inhibit of nucleic acid synthesis, Inhibite the enzyme dihydrofolate reductase	[123,124,125,126]
		Berberine		
		Hydrastine		
*Hyptis suaveolens*	EtOH extract	Diterpene, Indomethacin Suaveolol	NO, PGE2, SH compounds	[127]
*Impatiens balsamina*	Pod acetone, EtoAc, terpenoid fraction	2Methoxy1,4naphthoquinone	Produce ROS to damage *H pylori* cell membrane	[9]
		Stigmasta7,22-diene3βol		
*Ixeris chinensis*	Boiling water,EtOH and CHCl_3_ extract was the active one	Not identified	Antibacterial	[128]
			Antiadhesive	
			Anti-inflammatory	
			Inhibit IL-8, NO, TNF-α	
*Jatropha isabelli*	Acidified EtOH	Monoterpene	Gastroprotective	[129]
		1,4-Epoxy-ρ-menthan- 2-ol		
		Sesquiterpene		
		Cyperenoic acid		
		Triterpene		
		Acetyl aleuritolic acid		
		9b,13a- Dihydroxyisabellione		
		Diterpene		
		Jatropholone A		
		Jatropholone B Jatrophone		
*Juglans regia*	Fruit MeOH extract	Xanthanolide	Not detected	[130]
*Larrea divaricata*	Branches and leaves aqueous extract	Nordihydroguaiaretic acid	Anti-inflammatory	[131]
			Gastroprotective	
			Anti-gastric cancer	
*Lycopodium cernua*	Whole plant hexane extract	The powerful compound was found in hexane fraction	Not detected	[48]
*Magnoliae officinalis*	Ether fraction of cortex	Magnolol	Antigastritic, antioxidant, neutralize acid, inhibit the secretion of gastric acid	[132]
*Mallotus phillipinesis*	70 % EtOH extract of fruit	Isorottlerin, rottlerin	Not detected	[97]
		3′-prenylrubranine, 5,7-dihydroxy-8-methyl-6-prenylflavanone		
*Malva sylvestris*	Inflorescence and leaves EtOH Extract	Not identified	Not detected	[65]
*Mangifera indica*	Pet-ether and EtOH extracts of leaves	Mangiferin	Gastroprotective Antisecretory, antioxidant	[133,134]
*Mentha piperita*	Leaves andstem aqueous extract	Essential oil	antisecretory,antioxidant, anti-inflammatory, and antiapoptotic actions	[61]
		Menthol		
*Mentha sp.*	EtOH extract	Monoterpene	Increase PGE2	[38,39]
		Indomethacin pyloric ligature	Antiapoptotic,Antioxidant	
		Menthol	Anti-inflammatory	
*Morus alba*	leaves EtOH extract	Steroid, Albosteroid	Antisecretory	[135,136]
		Pyloric ligature	Antioxidant	
*Mitrella kentii*	EtOH extract	Chalcone	Antiapoptotic, antioxidant	[137]
		Desmosdumotin C	Inhibit COX-2	
*Musa acuminata*	Crude flavonoids extract	Flavonoids	Increase mucus	[138,139]
		Leucocyanidin		
*Myristica fragrans*	MeOH extracts of seeds and aerial parts	Not identified	Gastroprotective	[97,140]
*Myroxylon peruiferum*	Isolated compound	Isoflavone	Inhibit NADH oxidation	[141]
		Cabreuvin		
*Myrtus communis*	Essential oil	Monoterpenes	Inhibit urease	[86,142]
*Olea europaea*	Leaves MeOH extract	Not identified	Increase gastric flora	[143]
			Reduce H. pylori	
*Ocimum sanctum*	Fixed oil	Not identified	Inhibit lipoxygenase	[144]
			Antisecretory	
			Histamine antagonistic	
*Origanum majorana L.*	Aerial parts MeOH extract	Phenolic compounds	Enhance protective host defence	[45]
*Oroxylum indicum*	Crude Flavone glycosides	7-*O*-methylchrysin, 5-hydroxy-749-dimethoxyflavone, oroxylin A, chrysin, and baicalein	Gastroprotective	[145, [146]
*Paeonia lactiflora*	Root lipid fraction	Lysophosphatidic acid Paeonol benzoic acid methyl gallate,1,2,3,4,6-penta- *O*-galloyl-β -D-glucopyranose	Increase PG E2 Decrease membrane integrity Inhibit urease Inhibit UreB (an adhesin)	[5,147]
*Panax ginseng*	Polysaccharides fraction	Galacturonic acid	Anti-adhesive	[148,149]
*Papaver somniferum*	Alkaloids	Porphine	Not detected	[150]
*Pausinystalia yohimbe*	Alkaloids	Yohimbine	Decrease ulcer	[44]
*Peperomia pellucida*	EtOH extract	Allylbenzene Dillapiole	Gastroprotective	[151]
*Persea americana*	MeOH extracts of leaf	Procyanidins	Inhibit urease	[61]
*Piper carpunya*	Flavonoids rich extract of the leaves	Vitexin Isovitexin Rhamnopyranosylvitexin Isoembigenin	Releasemyeloperoxidase Inhibite H+,K + ATPase activity *N*-Acetylation	[154]
*Piper multiplinervium*	Hydroxybenzoic acid prenylated derivative	3-farnesyl-2-hydroxybenzoic acid	Treat stomach aches	[155]
*Pistacia lentiscus*	Mastic gum	Triterpenic acids	Induce blebbing Cellular fragmentation Morphological abnormalities in H. pylori cells	[156,157,158,159]
*Plectranthus grandis*	EtOH extract	Diterpenes 3b-Hydroxy-3- deoxibarbatusin Barbatusin	K^+^ATP channel NO, TRPV1 channels	[160]
*Plumbago zeylanica*	EtOAc of rhizome	Naphthoquinone Plumbagin	Bactericidal activity	[58,161]
*Polygala cyparissias*	EtOH extract	Xantone	Anti-ulcer Gastroprotective	[162]
*Polygonum tinctorium*	Leaf juice	Tryptanthrin Kaempferol	decrease numbers of colonies in gerbils stomachs	[163]
*Polygala cyparissias*	EtOH extract	Sterol a-Spinasterol	Reduce percentage of lesion area Reduce ulcer index	[162]
*Potentilla fruticose*	Aqueous extracts of aerial part	Not identified	Antibacterial action	[164]
*Prunus dulcis*	Polyphenol-rich extracts of skin	Protocatechuic acid	Post gastric plus duodenal digestion	[165]
*Prumnopitys andina*	Acidified EtOH	Diterpene, acetic acid Ferruginol	PGE2 production Inhibit lipoperoxidation	[37]
*Psoralea corylifolia*	Seeds extract	Psoracorylifols	Antibacterial	[166]
*Pteleopsis suberosa*	MeOH extract of stem bark	Oleanane saponine Arjunglucoside I	Anti*vacA/cagA* positive and metronidazole-resistant strains	[167]
*Punica granatum*	EtOH, MeOH, BuOH and aqueous extracts from fruit peel	Phenolic compounds	Chang hydrophobicity of *H. pylori* cell surface	[130,168,169]
*Phyllanthus niruri*	Aqueous extracts of leaves	Ellagic acid Hydroxycinnamic acid	Damage *H.pylori* cell membrane	[103,152]
*Physalis alkekengi*	EtOAc extract of the aerial parts	Quercetin Physalindicanols A kaempferol Blumenol A	Antiinflammatory Antiulcer invivo Analgesic	[153]
*Qualea parviflora*	MeOH extract of bark	Triterpenes Saponins	Maintaine GSH levels Increase SH compounds Stimulate PGE2 synthesis	[170]
*Rabdosia trichocarpa*	MeOH extract from entire plants	Diterpene Trichorabdal A	Strong antibacterial action	[171]
*Rhei Rhizoma*	Rhizome	Emodin	Damage DNA *H. Pylori*	[30]
*Rheum palmatum*	Rhizome	Rhein	Inhibite *N-*acetyltransferase	[172]
*Rheum rhaponticum L.*	Root EtOH Extract	Not identified	Anti-inflammatory	[56]
*Rosmarinus officinalis*	Leaves MeOH extract	Not identified	Antiulcer, vasodilator Gastroprotective	[45]
*Rubus imperialis*	EtOH extract	Triterpene 2b,3b-19a-Trihydroxy ursolic acid	Not detected	[173]
*Rubus ulmifolius*	Leaves extract Flavonoids	Ellagic Kampferol	Reduce gastric PH Participate No and SH	[26]
*Ruta graveolens*	Aqueous EtOH extract of leaves	Polyphenols	Antioxidant Anti-inflammatory Inhibit IL-8 secretion	[46]
*Salvia mirzayanii*	MeOH extract of leaves	Not identified	Not detected	[174]
*Sanguinaria Canadensis*	MeOH extracts of rhizome	Sanguinarine, chelerythrine, two benzophenanthridine alkaloids	Anti ulcer	[123,175]
*Santalum album*	hydro-alcoholic extract of stem	(Z)-R-santalol (7), (Z)-β-santalol, (Z)-lanceol	Strong antiulcer	[176]
*Schinus molle*	EtOH extract	Flavonol, Rutin	Antioxidant	[177]
*Sclerocarya birrea*	Essential oil	Terpinen- 4-ol	Decrease membrane integrity	[110,178]
*Senecio brasiliensis*	Inflorescences	Integerrimine, retrorsine, senecionine, usaramine, and seneciphylline	Increase mucus	[42,43]
	Pyrrolizidine alkaloids		Increase PG	
*Simaba ferruginea*	Rhizome fractions	Alkaloid	Antiulcerogenic	[41]
		Canthin-6-one	Reduce myeloperoxidase malondialdehyde	
			Reduce plasma IL-8	
*Scleria striatinux*	MeOH extract of roots	Okundoperoxide	Antibacterial	[48]
*Solanum paniculatum L.*	New isolated steroids saponins	diosgenin 3-*O*-b-d-glucopyranosyl(10 → 69)-*O*-b-d-glucopyranoiside.	Decrease gastric lesion	[179]
			Decrease levels of MPO in the mucosa	
*Sphacele chamaedryoide*	EtOH extract Diterpene	Horminone, Carnosol	Gastroprotective	[180]
		Taxoquinone	Inhibit gastric lesions	
*Stachys setifera*	MeOH extracts of leaves	Not identified	Not detected	[181]
*Strychnos pseudoquina*	Leaves MeOH extract	Alkaloid enriched fraction	Increase cell proliferation in gastric mucosa	[182]
*Syzygium aromaticum*	Flower buds	Flavonoids	Antiulcerogenic	[183,184]
		Tannins	Antisecretory	
			Increase PGE	
*Tabebuia impetiginosa*	Inner bark	(hydroxymethyl)anthraquin	Strong antibacterial	[185]
		anthraquinone-2-carboxylic		
		Lapachol, plumbagin		
*Termitomyces eurhizus*	Mushroom	Polysaccharides fraction	Stimulate mucosal regeneration and proliferation	[186]
			Restoring gastric mucus	
			Increase PG E2	
			Modulate COX-1 and COX-2	
			Reduce TNF-α and IL-1b	
*Terminalia spinosa*	Young branches crude extract	Not identified	Not detected	[187]
*Terminalia chebula*	Aqueous extracts of fruit	Chebulinic acid	Improve secretory of B runner gland	[188,189,190]
		Ethyl gallate gallic acid		
*Thymus vulgaris*	Essential oils	Monoterpenes	Gastroprotective	[191]
			Anti-inflammatory	
*Tithonia diversifolia*	EtOH extract	Sesquiterpene	Gastroprotective	[192]
		Indomethacin, Tagitinin C		
*Trachyspermum copticum*	Mixture of petroleum / MeOH extract of fruit and leaves	Not identified	Antibacterial	[78,193]
*Vaccinium macrocarpon*	Cranberry juice	Polyphenols	Anti-adhesive	[194,195]
*Vitis venefera*	Grape seeds	Resveratrol	Chemopreventative	[4]
	Flavonoids			
			Antioxidant	
*Xanthium brasilicum*	Aerial parts MeOH, diethyl ether and benzene	Not identified	Antimicrobial	[78]
*Zataria multiflora*	Essential oils of aerial parts	Thymol, carvacrol	Enhance mucosa Cytoprotective	[83,196]
*Zingiber officinalis*	Root extract	6-gingesulphonic acid	Inhibit thromboxane synthetase	[45,197,198,199,200,201,202]
		6-shogaol, Arcurcumene		
		Gingerols		

Methanol: MeOH; Ethanol: EtOH; Butanol: BuOH; Dichloromethan: CH_2_Cl_2;_ Chloroform:CHCl_3_; Prostaglandin: PG; Tumor necrosis factor: TNF; Interlokin: IL; Cyclooxiginase: COX; Nitric oxide: NO; sulfhydryl : SH.

### 4.1 Sterol

**Fig. 3:**
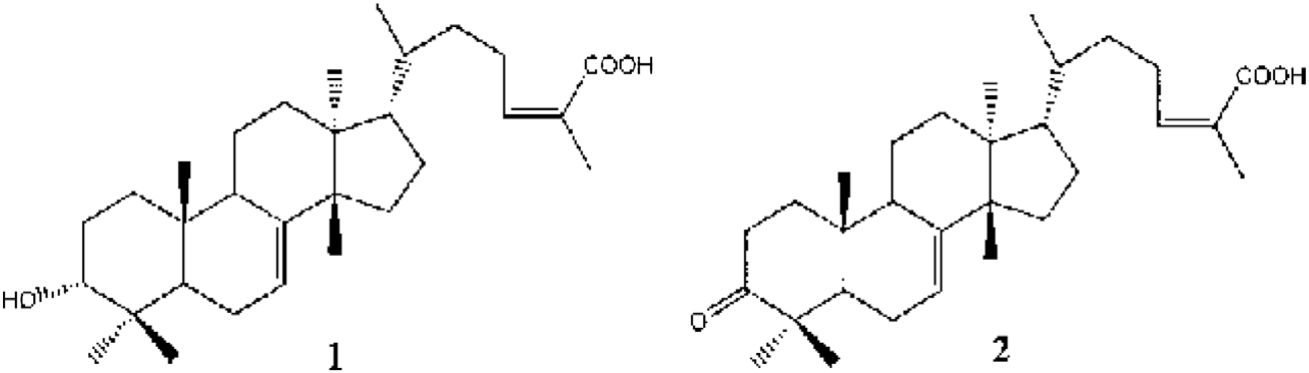
Chemical structure of 3a-hydroxymasticadienonic acid (1) and masticadienonic acid (2).

The presence of a free OH group in C-3 is necessary for the antiulcer action of triterpenoids and sterols consistently, the only structural difference between the active 3a-hydroxymasticadienonic acid (Fig. 3, 1) and the inactive masticadienonic acid (Fig. 3, 2) is the presence of an OH group and a C=O group in the C-3 [14,15].

### 4.2 Flavonoids

**Fig. 4:**
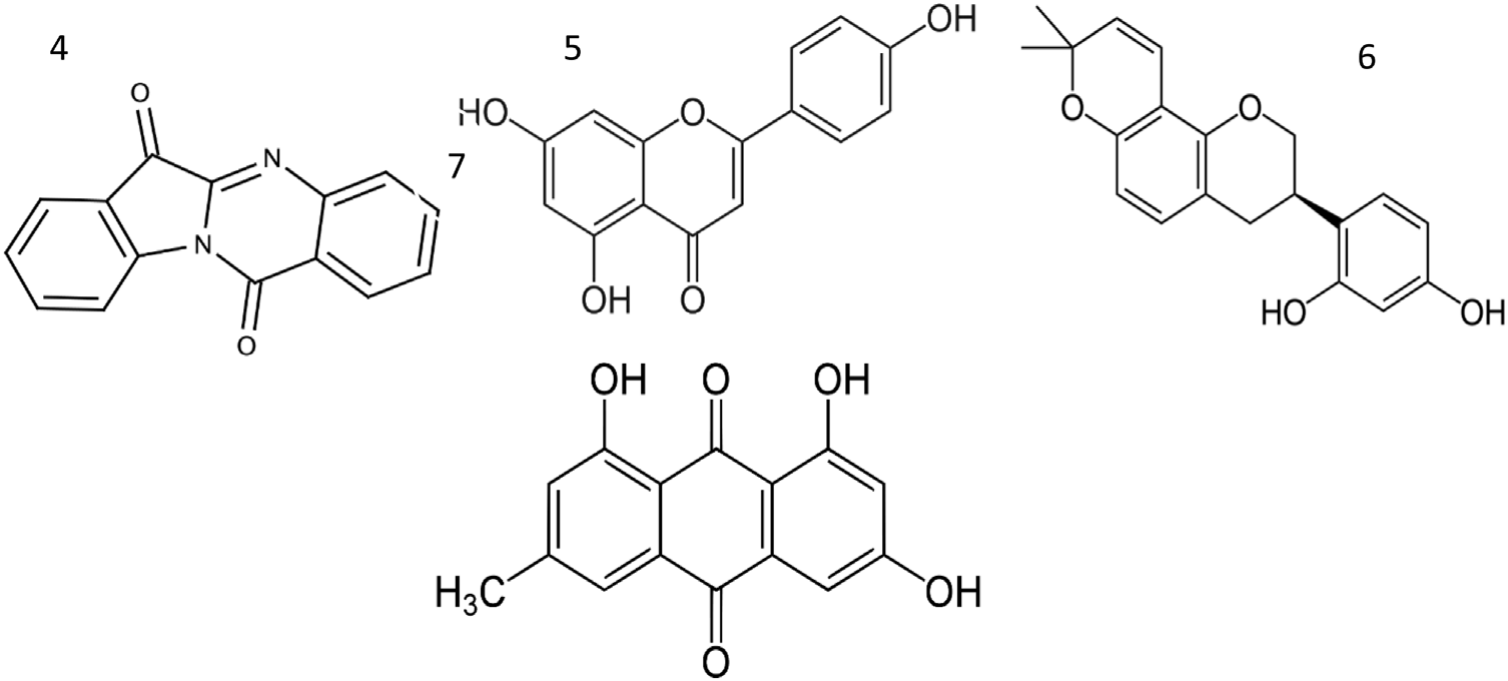
Chemical structure of anti-H.Pylori flavonoids 1) Quercetin 2) Kampferol 3) Catchin 4) tryptanthrin 5) Apigenin 6) Glabridin 7) Emodin.

Flavonoids have been used in the treatment of countless diseases [[16], [17], [18], [19], [20], [21]]. Flavonoids (Fig. 4) are found to display as antisecretory and cytoprotective agents by increasing PG levels, inhibiting *H. pylori*, decreasing histamine, and antioxidants [22]. The structure activity relationship shows that the presence of OCH_3_ group in the C-5 or C-7 positions, the double bonds at C-2 and C-3 and the presence pof an intact C-ring appear to increase gastroprotection potential. On the other hand, substitution with OH or OCH_3_ groups at C-3, C-6, or C-8 diminish the gastroprotective action.

Flavonoids can kill microbs by 1) membrane disruption by apigenin, catechin, naringenin, quercetin, and rhamnetin and inhibition of nucleic acid synthesis 2) inhibit dihydrofolate reductase by epicatechin, 3) inhibithelicase by luteolin and myricetin, d) inhibitgyrase/topoisomerase by apigenin, kaempferol and quercetin, 4) inhibit bacterial virulence by quercetin and kaempferol 5) inhibit quorum sensing by epicatechin, naringenin, quercetin and kaempferol 6) inhibit fatty acid synthase and peptidoglycan synthesis by taxifolin, kaempferol, luteolin, myricetin and quercetin7) inhibit Ala–Ala dipeptide synthesis by gaiangin, kaempferol, and kaempferol-3-*O*-glucoside, 8) inhibitpeptidoglycan crosslinking by apigenin and quercetin. 9) inhibit refflux pumps by diadzein, genistein, epicatechin and quercetin10) inhibit NADH-cytochrome c reductase activity in the bacterial respiratory chain by chalcon11) inhibit ATP synthase by epicatchin, quercetin, quercetrin, and silymarin [23].

As shown in Fig. 4, quercetin decreases lipid peroxide and neutrophil leukocyte infiltration, in the *H. pylori* colonization [24]. The blend of kaempferol and tryptanthrin reduce the viability of *H. pylori* invivo [25,26]. Upon giving green tea product that is consisted of catechin to *H. pylori*-infected Mongolian gerbils, both of gastritis and the prevalence of *H. pylori* were significantly suppressed [27]. Besides, apigenin treatments effectively eradicated *H. pylori,* atrophic gastritis, and gastric cancer rates in *H. pylori*-infected Mongolian gerbils. Apigenin is reported to have excellent ability to inhibit *H. pylori* as well as possessing potent anti-gastric cancer [28]. As for Glabridin, it possesses a strong inhibitory effect on dihydrofolate reductase and DNA gyrase [29]. While emodin; a major phytocompound of *Rhizoma Rhei* induces *H. pylori* DNA damage [30].

### 4.3 Steroid saponin

**Fig. 5:**
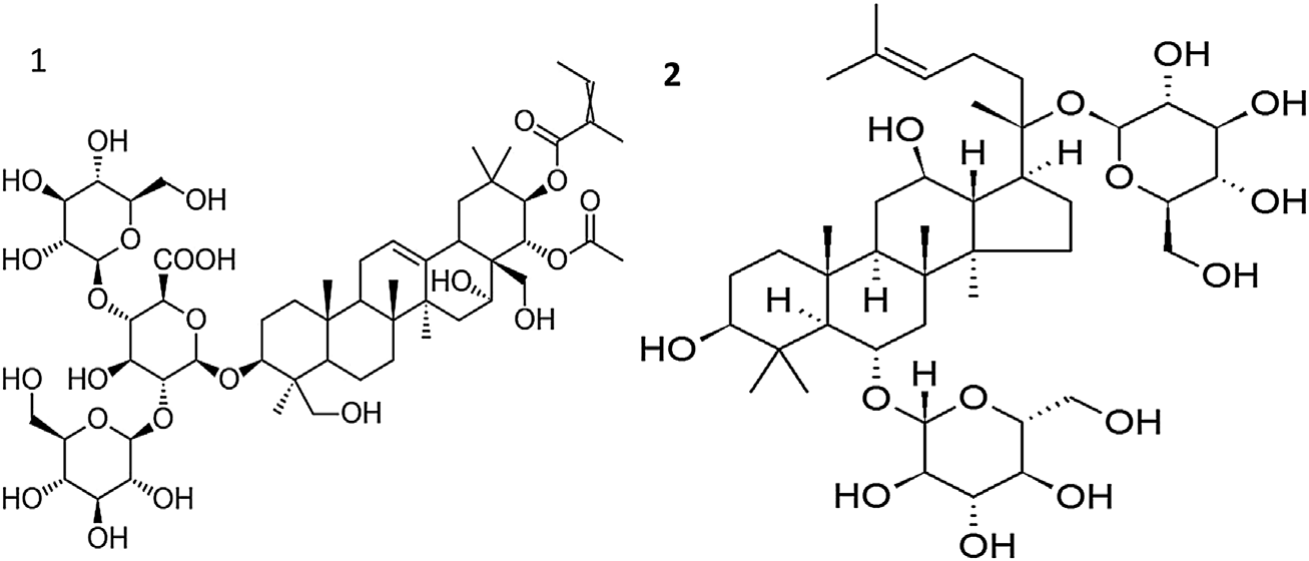
Chemical structure of Aescine (1) and Ginsenoside (2).

Aescine (Fig. 5) reduces the severity of ulcers by decreasing gastric secretion [31], while Ginsenoside increases the amount of mucus [32].

According to Lee et al. [33], the saponins display antisecretory action by inhibiting acid secretion, total acid output, and lowering the pH of gastric juice [34].

### 4.4 Terpenes

**Fig. 6:**
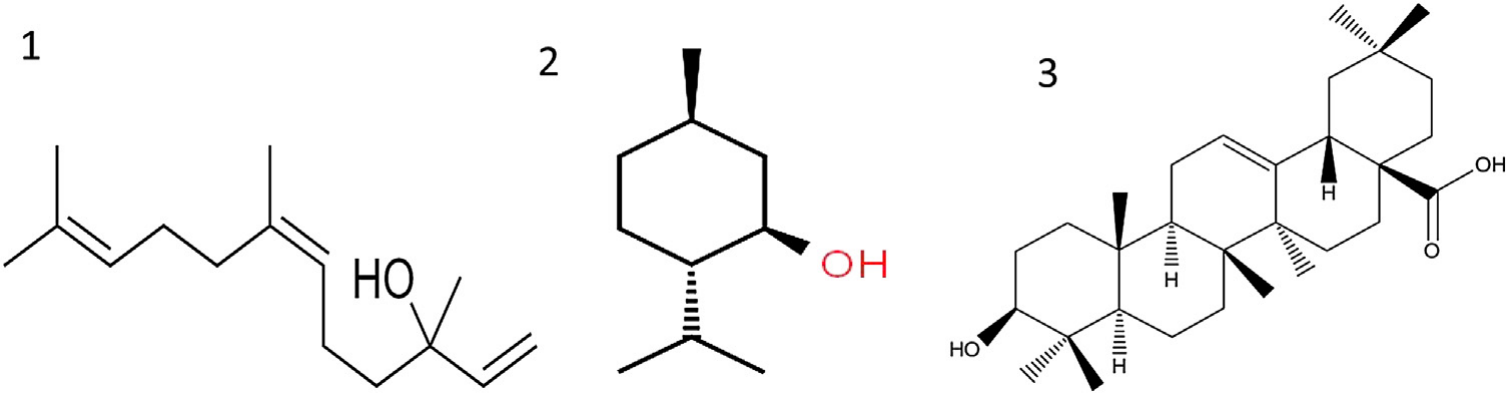
Chemical structure of anti-H.pylori terpens 1) Nerolidol 2) Menthol 3) Oleanolic acid.

Nerolidol (Fig. 6) has an antiulcerogenic and cytoprotective effect by increasing mucus production via increasing the PG, improving the gastric blood flow, and increasing the secretion of gastric bicarbonate and mucus [35]. In addition, terpenoids act as antioxidants, reduce the lipid peroxidation levels, and increase the activity of antioxidant enzymes in the gastric mucosa [36,37]. Menthol is a monoterpene that increases the maintenance of SH compounds and the amount of mucus and PG production. It also possesses an antisecretory effect, in addition to antioxidant, anti-inflammatory, and antiapoptotic actions [38,39]. Oleanolic acid is a triterpene that improves healing in the ulcer model. The low toxicity and the widespread occurrence in various plants support the potential development of new antiulcer drug based on triterpenes or their derivatives [37].

### 4.5 Polysaccharides

**Fig. 7:**
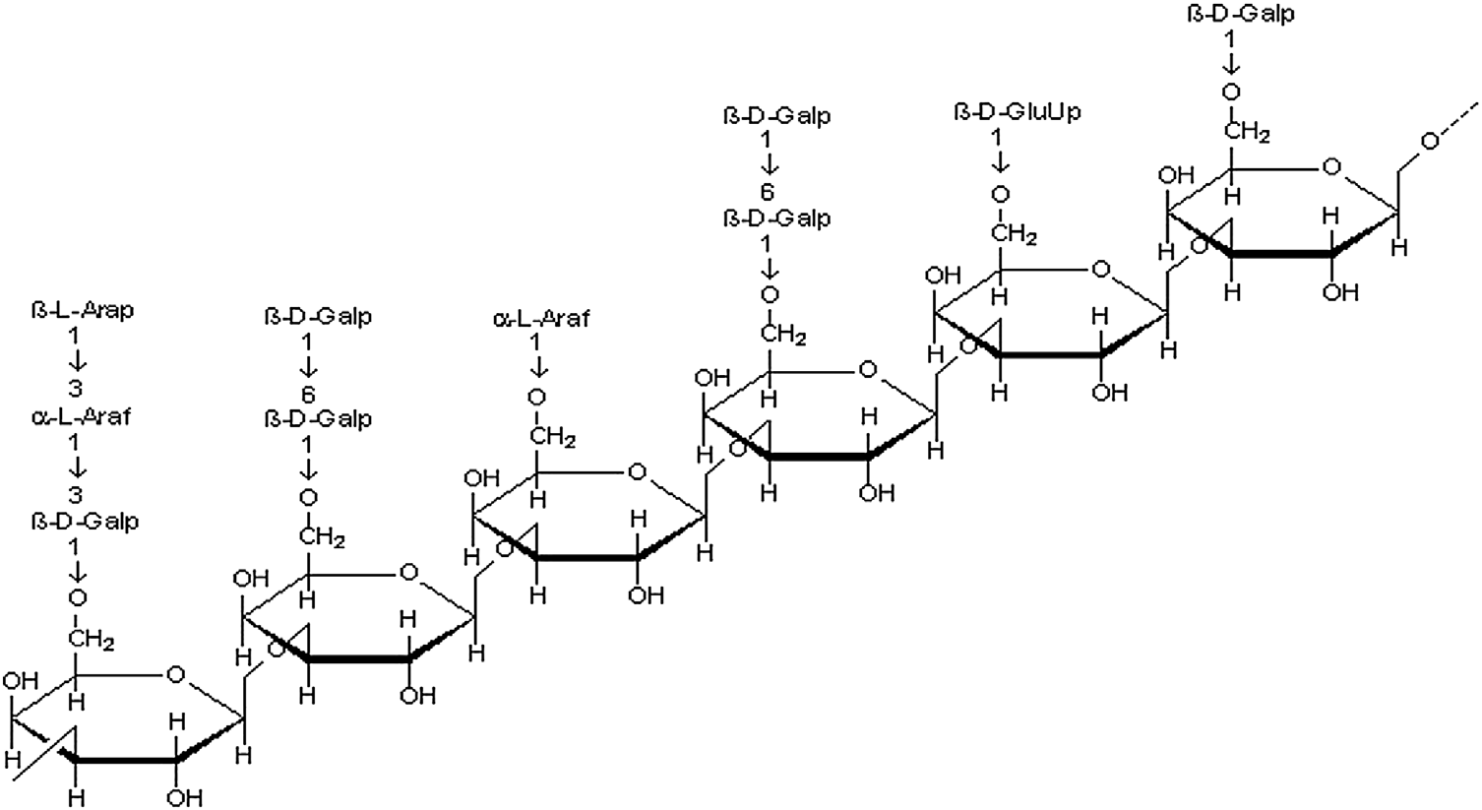
chemical structure of Arabinogalactan.

Arabinogalactan (Fig. 7) has the ability to bind on the gastric mucosa acting as a protective layer, in addition to its antisecretory activity towards gastric juice. The mucosal protective activity of Arabinogalactan is provided by an increased mucus synthesis and free radical scavenging activity. The particular mechanisms of polysaccharides are described by their potential to bind on the surface of the gastrointestinal mucosa, thereby acting as a protective layer, in addition to their antisecretory action. Their mucosal protective potentials are provided by an increased mucus synthesis and their antioxidant activity. Pectic polysaccharides obtained by aqueous extraction represent examples of the main polysaccharides displaying gastric antiulcer action [40].

### 4.6 Alkaloids

**Fig. 8:**
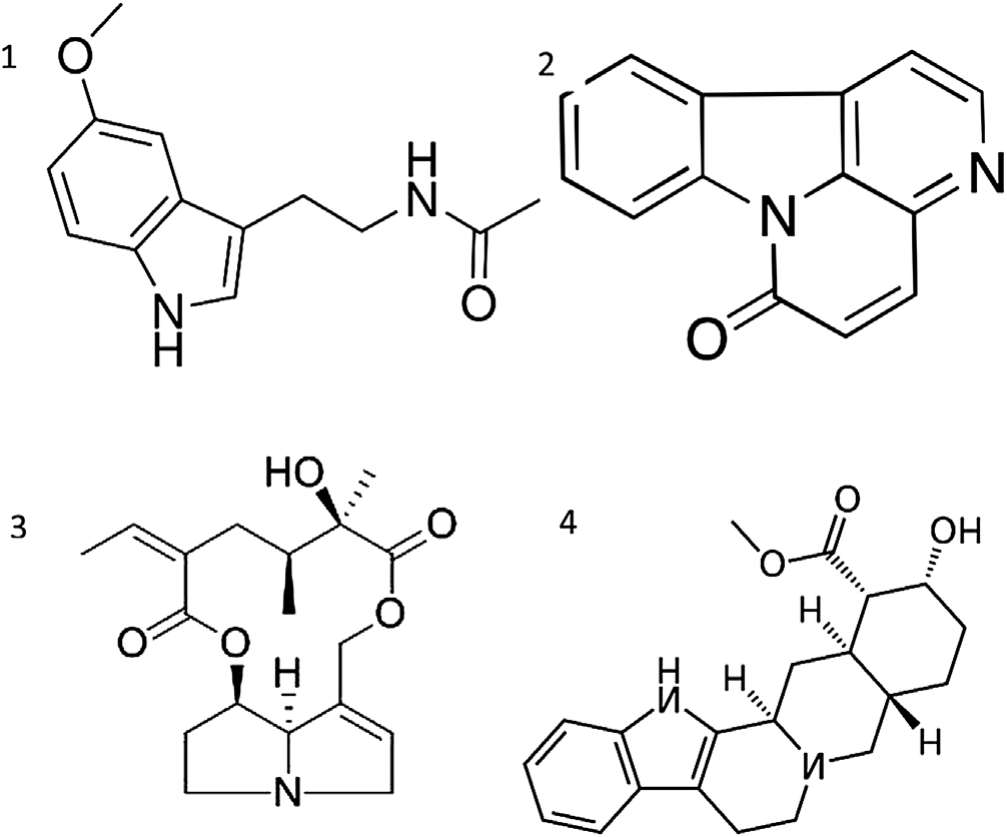
Chemical structure of Melatonin (1), Canthin-6-one (2), Integerrimine (3), Yohimbine (4).

Canthin-6-one (Fig. 8), isolated from *Simaba ferruginea* rhizome has been shown to be antiulcerogenic [41], while integerrimine isolated from *Senecio brasiliensis* was found to increase mucus and PG levels [42,43]. Melatonin, as a hormone, has the ability to scavenge free radical and ameliorating gastric blood flow [43]. Yohimbine, isolated from *Pausinystalia yohimbe*, decreases ulcers [44].

## 5 Conclusion

*H. pylori* inhibition with antibiotic therapies has a limitation mainly owing to antibiotic resistance. Medicinal herbs provide another opportunity to inhibit *H. pylori*. Medicinal herbs might also provide successful approach to decrease stomach cancer. However, potential cytotoxicity and side effects might present from those herbs. Therefore, further cytotoxicity investigation will be required.

## Declaration of Competing Interest

The authors declare that they have no known competing financial interests or personal relationships that could have appeared to influence the work reported in this paper.

## References

[1] Lawal T.O., Igbokwe C.O., Adeniyi B.A. (2014). Antimicrobial activities and the bactericidal kinetics of *Allium ascalonicum* Linn. whole plant) against standard and clinical strains of *Helicobacter pylori*: support for ethnomedical use. J. Nat. Sci. Res..

[2] Conteduca V., Sansonno D., Lauletta G., Russi S., Ingravallo G., Dammacco F. (2013). *H. pylori* infection and gastric cancer: state of the art. Int. J. Oncol..

[3] Wolle K., Malfertheiner P. (2007). Treatment of *Helicobacter pylori*. Best Pract. Res. Clin. Gastroenterol..

[4] Paulo L., Oleastro M., Gallardo E., Queiroz J.A., Domingues F. (2011). Anti-*Helicobacter pylori* and urease inhibitory activities of resveratrol and red wine. Food Res. Int..

[5] Ngan L.T.M., Moon J.K., Shibamoto T., Ahn Y.J. (2012). Growth-inhibiting, bactericidal, and urease inhibitory effects of *Paeonia lactiflora* root constituents and related compounds on antibiotic-susceptible and-resistant strains of *Helicobacter pylori*. J. Agric. Food Chem..

[6] do Carmo Souza M., Beserra A.M.S., Martins D.C., Real V.V., dos Santos R.A.N., Rao V.S., da Silva R.M., de Oliveira Martins D.T. (2009). In vitro and in vivo anti-Helicobacter pylori activity of *Calophyllum brasiliense* Camb. J. Ethnopharmacol..

[7] Lin Y.T., Kwon Y.I., Labbe R.G., Shetty K. (2005). Inhibition of *Helicobacter pylori* and associated urease by oregano and cranberry phytochemical synergies. Appl. Environ. Microbiol..

[8] Amin M., Anwar F., Naz F., Mehmood T., Saari N. (2013). Anti-*Helicobacter pylori* and urease inhibition activities of some traditional medicinal plants. Molecules.

[9] Wang Y.C., Li W.Y., Wu D.C., Wang J.J., Wu C.H., Liao J.J., Lin C.K. (2011). In vitro activity of 2-methoxy-1, 4-naphthoquinone and stigmasta-7, 22-diene-3β-ol from *Impatiens balsamina* L. against multiple antibiotic-resistant *Helicobacter pylori*. Evid. Based Complement. Altern. Med..

[10] O’Mahony R., Al-Khtheeri H., Weerasekera D., Fernando N., Vaira D., Holton J., Basset C. (2005). Bactericidal and anti-adhesive properties of culinary and medicinal plants against *Helicobacter pylori*. World J. Gastroenterol..

[11] Wittschier N., Faller G., Hensel A. (2009). Aqueous extracts and polysaccharides from liquorice roots (*Glycyrrhiza glabra* L.) inhibit adhesion of *Helicobacter pylori* to human gastric mucosa. J. Ethnopharmacol..

[12] Wittschier N., Faller G., Hensel A. (2007). An extract of Pelargonium sidoides (EPs 7630) inhibits in situ adhesion of *Helicobacter pylori* to human stomach. Phytomedicine.

[13] Takagi A., Koga Y., Aiba Y., Kabir A.M., Watanabe S., Ohta‐Tada U., Osaki T., Kamiya S., Miwa T. (2000). Plaunotol suppresses interleukin‐8 secretion induced by *Helicobacter pylori*: therapeutic effect of plaunotol on *H. pylori* infection. J. Gastroenterol. Hepatol..

[14] Giner-Larza E.M., Máñez S., Giner R.M., Recio M.C., Prieto J.M., Cerdá-Nicolás M., Ríos J. (2002). Anti-inflammatory triterpenes from *Pistacia terebinthus* galls. Planta Med..

[15] Navarrete A., Trejo-Miranda J.L., Reyes-Trejo L. (2002). Principles of root bark of Hippocratea excelsa (Hippocrataceae) with gastroprotective activity. J. Ethnopharmacol..

[16] El-Gengaihi S.E., Hamed M.A., Aboubaker D.H., Mossa A.T. (2016). Flavonoids from sugar beet leaves as hepatoprotective agent. Int. J. Pharm. Pharm. Sci..

[17] El-Gengaihi S.E., Mossa A.T.H., Refaie A.A., Aboubaker D. (2016). Hepatoprotective efficacy of *Cichorium intybus* L. extract against carbon tetrachloride-induced liver damage in rats. J. Diet. Suppl..

[18] Baker D.A., Al-Moghazy M., ElSayed A.A.A. (2020). The in vitro cytotoxicity, antioxidant and antibacterial potential of *Satureja hortensis* L. essential oil cultivated in Egypt. Bioorg. Chem..

[19] Abou Baker D.H., Rady Hanaa M. (2020). Bioassay-guided approach employed to isolate and identify anticancer compounds from *Physalis peruviana* calyces. Plant Arch..

[20] Abou Baker D.H. (2020). *Achillea millefolium* L. ethyl acetate fraction induces apoptosis and cell cycle arrest in human cervical cancer (HeLa) cells. Ann. Agric. Sci..

[21] Salam M.A., Ibrahim B.M., El-Batran S.E., El-Gengaihi S.E., Baker D.H. (2016). Study of the possible antihypertensive and hypolipidemic effects of an herbal mixture on l-name-induced hypertensive rats. Asian J. Pharm. Clin. Res..

[22] Coelho R.G., Batista L.M., Santos L.C.D., Brito A.R.M.D.S., Vilegas W. (2006). Phytochemical study and antiulcerogenic activity of *Syngonanthus bisulcatus* (Eriocaulaceae). Rev. Bras. Cinêc. Farm..

[23] Olaleye S.B., Farombi E.O. (2006). Attenuation of indomethacin‐and HCl/ethanol‐induced oxidative gastric mucosa damage in rats by kolaviron, a natural biflavonoid of *Garcinia kola* seed. Phytother. Res..

[24] González-Segovia R., Quintanar J.L., Salinas E., Ceballos-Salazar R., Aviles-Jiménez F., Torres-López J. (2008). Effect of the flavonoid quercetin on inflammation and lipid peroxidation induced by *Helicobacter pylori* in gastric mucosa of guinea pig. J. Gastroenterol..

[25] Kataoka M., Hirata K., Kunikata T., Ushio S., Iwaki K., Ohashi K., Ikeda M., Kurimoto M. (2001). Antibacterial action of tryptanthrin and kaempferol, isolated from the indigo plant (*Polygonum tinctorium* Lour.), against *Helicobacter pylori*-infected Mongolian gerbils. J. Gastroenterol..

[26] Martini S., D’Addario C., Colacevich A., Focardi S., Borghini F., Santucci A., Figura N., Rossi C. (2009). Antimicrobial activity against *Helicobacter pylori* strains and antioxidant properties of blackberry leaves (*Rubus ulmifolius*) and isolated compounds. Int. J. Antimicrob. Agents.

[27] Matsubara S., Shibata H., Ishikawa F., Yokokura T., Takahashi M., Sugimura T., Wakabayashi K. (2003). Suppression of *Helicobacter pylori*-induced gastritis by green tea extract in Mongolian gerbils. Biochem. Biophys. Res. Commun..

[28] Kuo C.H., Weng B.C., Wu C.C., Yang S.F., Wu D.C., Wang Y.C. (2014). Apigenin has anti-atrophic gastritis and anti-gastric cancer progression effects in *Helicobacter pylori*-infected Mongolian gerbils. J. Ethnopharmacol..

[29] Asha M.K., Debraj D., Edwin J.R., Srikanth H.S., Muruganantham N., Dethe S.M., Anirban B., Jaya B., Deepak M., Agarwal A. (2013). In vitro anti-*Helicobacter pylori* activity of a flavonoid rich extract of *Glycyrrhiza glabra* and its probable mechanisms of action. J. Ethnopharmacol..

[30] Wang H.H., Chung J.G. (1997). Emodin-induced inhibition of growth and DNA damage in the *Helicobacter pylori*. Curr. Microbiol..

[31] Marhuenda E., Martin M.J., Alarcon Lastra C.D.L. (1993). Antiulcerogenic activity of aescine in different experimental models. Phytother. Res..

[32] Jeong C.S., Hyun J.E., Kim Y.S., Lee E.S. (2003). Ginsenoside RB 1 the anti-ulcer constituent from the head of *Panax ginseng*. Arch. Pharm. Res..

[33] Lee E.B., Kim O.J., Kang S.S., Jeong C. (2005). Araloside A, an antiulcer constituent from the root bark of *Aralia elata*. Biol. Pharm. Bull..

[34] Klopell F.C., Lemos M., Sousa J.P.B., Comunello E., Maistro E.L., Bastos J.K., De Andrade S.F. (2007). Nerolidol, an antiulcer constituent from the essential oil of *Baccharis dracunculifolia* DC (Asteraceae). Z. Naturforschung C.

[35] Ohta Y., Kamiya Y., Imai Y., Arisawa T., Nakano H. (2005). Plaunotol prevents the progression of acute gastric mucosal lesions induced by compound 48/80, a mast cell degranulator, in rats. Pharmacology.

[36] Kim J.H., Kim Y.S., Song G.G., Park J.J., Chang H.I. (2005). Ulcers and gastrointestinal health. Eur. J. Pharmacol..

[37] Rodríguez J.A., Theoduloz C., Yáñez T., Becerra J., Schmeda-Hirschmann G. (2006). Gastroprotective and ulcer healing effect of ferruginol in mice and rats: assessment of its mechanism of action using in vitro models. Life Sci..

[38] Rozza A.L., Hiruma-Lima C.A., Takahira R.K., Padovani C.R., Pellizzon C.H. (2013). Effect of menthol in experimentally induced ulcers: pathways of gastroprotection. Chem. Biol. Interact..

[39] Rozza A.L., de Faria F.M., Brito A.R.S., Pellizzon C.H. (2014). The gastroprotective effect of menthol: involvement of anti-apoptotic, antioxidant and anti-inflammatory activities. PLoS One.

[40] Nergard C.S., Diallo D., Inngjerdingen K., Michaelsen T.E., Matsumoto T., Kiyohara H., Yamada H., Paulsen B.S. (2005). Medicinal use of *Cochlospermum tinctorium* in Mali: anti-ulcer-, radical scavenging-and immunomodulating activities of polymers in the aqueous extract of the roots. J. Ethnopharmacol..

[41] Almeida E.S.S., Filho V.C., Niero R., Clasen B.K., Balogun S.O., Oliveira Martins D.T. (2011). Pharmacological mechanisms underlying the anti-ulcer activity of methanol extract and canthin-6-one of *Simaba ferruginea* A. St-Hil. in animal models. J. Ethnopharmacol..

[42] Toma W., Trigo J.R., Bensuaski de Paula A.C., Souza Brito (2004). ARM Preventive activity of pyrrolizidine alkaloids from *Senecio brasiliensis* (Asteraceae) on gastric and duodenal induced ulcer on mice and rats. J. Ethnopharmacol..

[43] Konturek P.C., Konturek S.J., Majka J., Zembala M., Hahn E.G. (1997). Melatonin affords protection against gastric lesions induced by ischemia-reperfusion possibly due to its antioxidant and mucosal microcirculatory effects. Eur. J. Pharmacol..

[44] Ozaki Y. (1989). Pharmacological studies of indole alkaloids obtained from domestic plants, *Uncaria rhynchophylla* Miq. and Amsonia elliptica Roem. Et Schult. *Nihon yakurigaku zasshi*. Folia Pharmacol. Jpn..

[45] Mahady G.B., Pendland S.L., Stoia A., Hamill F.A., Fabricant D., Dietz B.M., Chadwick L.R. (2005). In vitro susceptibility of *Helicobacter pylori* to botanical extracts used traditionally for the treatment of gastrointestinal disorders. Phytother. Res..

[46] Zaidi S.F., Muhammad J.S., Shahryar S., Usmanghani K., Gilani A.H., Jafri W., Sugiyama T. (2012). Anti-inflammatory and cytoprotective effects of selected Pakistani medicinal plants in *Helicobacter pylori*-infected gastric epithelial cells. J. Ethnopharmacol..

[47] Sánchez-Mendoza M.E., Rodríguez-Silverio J., Rivero-Cruz J.F., Rocha-González H.I., Pineda-Farías J.B., Arrieta J. (2013). Antinociceptive effect and gastroprotective mechanisms of 3, 5-diprenyl-4-hydroxyacetophenone from *Ageratina pichinchensis*. Fitoterapia.

[48] Ndip R.N., Tarkang A.E.M., Mbullah S.M., Luma H.N., Malongue A., Ndip L.M., Nyongbela K., Wirmum C., Efange S.M. (2007). In vitro anti-*Helicobacter pylori* activity of extracts of selected medicinal plants from North West Cameroon. J. Ethnopharmacol..

[49] Li H., Meng L., Liu F., Wei J.F., Wang Y.Q. (2013). H+/K+-ATPase inhibitors: a patent review. Expert Opin. Ther. Pat..

[50] Lima Z.P., Calvo T.R., Silva E.F., Pellizzon C.H., Vilegas W., Brito A.R.M.S., Bauab T.M., Hiruma-Lima C.A. (2008). Brazilian medicinal plant acts on prostaglandin level and *Helicobacter pylori*. J. Med. Food.

[51] Lima Z.P., Bonamin F., Calvo T.R., Vilegas W., Santos L.C., Rozza A.L., Pellizzon C.H., Rocha L.R., Hiruma-Lima C.A. (2011). Effects of the ethyl acetate fraction of *Alchornea triplinervia* on healing gastric ulcer in rats. Pharmaceuticals.

[52] O’Gara E.A., Hill D.J., Maslin D.J. (2000). Activities of garlic oil, garlic powder, and their diallyl constituents against *Helicobacter pylori*. Appl. Environ. Microbiol..

[53] Gail M.H., Pfeiffer R.M., Brown L.M., Zhang L., Ma J.L., Pan K.F., Liu W.D., You W.C. (2007). Garlic, vitamin, and antibiotic treatment for *Helicobacter pylori*: a randomized factorial controlled trial. Helicobacter.

[54] Gu L.K., Zhou P., Zhou J., Wang R.M., Yang W.J., Deng D.J. (2007). Effect of selenium-enriched garlic on chronic gastritis of the glandular stomach of *Mongolian gerbils* induced by *H. pylori*. Zhonghua Yu Fang Yi Xue Za Zhi.

[55] Liu S., Sun Y., Li W., Yu H., Li X., Liu Z., Zeng J., Zhou Y., Chen C., Jia J. (2010). The antibacterial mode of action of allitridi for its potential use as a therapeutic agent against *Helicobacter pylori* infection. FEMS Microbiol. Lett..

[56] Cellini L., Di Campli E., Masulli M., Di Bartolomeo S., Allocati N. (1996). Inhibition of *Helicobacter pylori* by garlic extract (*Allium sativum*). FEMS Immunol. Med. Microbiol..

[57] Prabjone R., Thong-Ngam D., Wisedopas N., Chatsuwan T., Patumraj S. (2006). Anti-inflammatory effects of *Aloe vera* on leukocyte–endothelium interaction in the gastric microcirculation of *Helicobacter pylori*-infected rats. Clin. Hemorheol. Microcirc..

[58] Hsieh S.C., Fang S.H., Rao Y.K., Tzeng Y.M. (2008). Inhibition of pro-inflammatory mediators and tumor cell proliferation by *Anisomeles indica* extracts. J. Ethnopharmacol..

[59] Rosas-Acevedo H., Terrazas T., González-Trujano M.E., Guzmán Y., Soto-Hernández M. (2011). Anti-ulcer activity of *Cyrtocarpa procera* analogous to that of Amphipterygium adstringens, both assayed on the experimental gastric injury in rats. J. Ethnopharmacol..

[60] Cho C.H., Mei Q.B., Shang P., Lee S.S., So H.L., Guo X., Li Y. (2000). Study of the gastrointestinal protective effects of polysaccharides from *Angelica sinensis* in rats. Planta Med..

[61] Castillo-Juárez I., González V., Jaime-Aguilar H., Martínez G., Linares E., Bye R., Romero I. (2009). Anti-*Helicobacter pylori* activity of plants used in Mexican traditional medicine for gastrointestinal disorders. J. Ethnopharmacol..

[62] Konstantinopoulou M., Karioti A., Skaltsas S., Skaltsa H. (2003). Sesquiterpene lactones from *Anthemis altissima* and their anti-*Helicobacter pylori* activity. J. Nat. Prod..

[63] Mafioleti L., da Silva Junior I.F., Colodel E.M., Flach A., de Oliveira Martins D.T. (2013). Evaluation of the toxicity and antimicrobial activity of hydroethanolic extract of *Arrabidaea chica* (Humb. & Bonpl.) B. Verl. J. Ethnopharmacol..

[64] Wang K.T., Chen L.G., Wu C.H., Chang C.C., Wang C.C. (2010). Gastroprotective activity of atractylenolide III from *Atractylodes ovata* on ethanol‐induced gastric ulcer in vitro and in vivo. J. Pharm. Pharmacol..

[65] Cogo L.L., Monteiro C.L.B., Miguel M.D., Miguel O.G., Cunico M.M., Ribeiro M.L., Camargo E.R.D., Kussen G.M.B., Nogueira K.D.S., Costa L.M.D. (2010). Anti-*Helicobacter pylori* activity of plant extracts traditionally used for the treatment of gastrointestinal disorders. Braz. J. Microbiol..

[66] Abdelwahab S.I., Mohan S., Abdulla M.A., Sukari M.A., Abdul A.B., Taha M.M.E., Syam S., Ahmad S., Lee K.H. (2011). The methanolic extract of *Boesenbergia rotunda* (L.) Mansf. and its major compound pinostrobin induces anti-ulcerogenic property in vivo: possible involvement of indirect antioxidant action. J. Ethnopharmacol..

[67] Epifano F., Menghini L., Pagiotti R., Angelini P., Genovese S., Curini M. (2006). In vitro inhibitory activity of boropinic acid against *Helicobacter pylori*. Bioorg. Med. Chem. Lett..

[68] Galan M.V., Kishan A.A., Silverman A.L. (2004). Oral broccoli sprouts for the treatment of *Helicobacter pylori* infection: a preliminary report. Dig. Dis. Sci..

[69] Banskota A.H., Tezuka Y., Adnyana I.K., Ishii E., Midorikawa K., Matsushige K., Kadota S. (2001). Hepatoprotective and anti-*Helicobacter pylori* activities of constituents from Brazilian propolis. Phytomedicine.

[70] Okeleye B.I., Bessong P.O., Ndip R.N. (2011). Preliminary phytochemical screening and in vitro anti-*Helicobacter pylori* activity of extracts of the stem bark of Bridelia micrantha (Hochst., Baill., Euphorbiaceae). Molecules.

[71] Adefuye A.O., Ndip R.N. (2013). Phytochemical analysis and antibacterial evaluation of the ethyl acetate extract of the stem bark of *Bridelia micrantha*. Pharmacogn. Mag..

[72] Bonacorsi C., Da Fonseca L.M., Raddi M.S.G., Kitagawa R.R., Vilegas W. (2013). Comparison of Brazilian plants used to treat gastritis on the oxidative burst of *Helicobacter pylori*-stimulated neutrophil. Evid. Based Complement. Altern. Med..

[73] Lima Z.P., dos Santos R.D.C., Torres T.U., Sannomiya M., Rodrigues C.M., dos Santos L.C., Pellizzon C.H., Rocha L.R.M., Vilegas W., Brito A.R.M.S., Cardoso C.R.P. (2008). Byrsonima fagifolia: an integrative study to validate the gastroprotective, healing, antidiarrheal, antimicrobial and mutagenic action. J. Ethnopharmacol..

[74] Santos R.C., Kushima H., Rodrigues C.M., Sannomiya M., Rocha L.R.M., Bauab T.M., Tamashiro J., Vilegas W., Hiruma-Lima C.A. (2012). Byrsonima intermedia A. Juss.: gastric and duodenal anti-ulcer, antimicrobial and antidiarrheal effects in experimental rodent models. J. Ethnopharmacol..

[75] Lemos L.M.S., Martins T.B., Tanajura G.H., Gazoni V.F., Bonaldo J., Strada C.L., da Silva M.G., Dall’Oglio E.L., de Sousa Júnior P.T., de Oliveira Martins D.T. (2012). Evaluation of antiulcer activity of chromanone fraction from *Calophyllum brasiliesnse* Camb. J. Ethnopharmacol..

[76] Takabayashi F., Harada N., Yamada M., Murohisa B., Oguni I. (2004). Inhibitory effect of green tea catechins in combination with sucralfate on *Helicobacter pylori* infection in Mongolian gerbils. J. Gastroenterol..

[77] Ruggiero P., Rossi G., Tombola F., Pancotto L., Lauretti L., Del Giudice G., Zoratti M. (2007). Red wine and green tea reduce *H pylori*-or VacA-induced gastritis in a mouse model. World J. Gastroenterol.: WJG.

[78] Nariman F., Eftekhar F., Habibi Z., Massarrat S., Malekzadeh R. (2009). Antibacterial activity of twenty Iranian plant extracts against clinical isolates of *Helicobacter pylori*. Iran. J. Basic Med. Sci..

[79] Spósito L., Oda F.B., Vieira J.H., Carvalho F.A., dos Santos Ramos M.A., de Castro R.C., Crevelin E.J., Crotti A.E.M., Santos A.G., da Silva P.B., Chorilli M. (2019). In vitro and in vivo anti-*Helicobacter pylori* activity of *Casearia sylvestris* leaf derivatives. J. Ethnopharmacol..

[80] Shikov A.N., Pozharitskaya O.N., Makarov V.G., Kvetnaya A.S. (2008). Antibacterial activity of *Chamomilla recutita* oil extract against *Helicobacter pylori*. Phytother. Res..

[81] Stamatis G., Kyriazopoulos P., Golegou S., Basayiannis A., Skaltsas S., Skaltsa H. (2003). In vitro anti-*Helicobacter pylori* activity of Greek herbal medicines. J. Ethnopharmacol..

[82] Mabe K., Yamada M., Oguni I., Takahashi T. (1999). In vitro and in vivo activities of tea catechins against *Helicobacter pylori*. Antimicrob. Agents Chemother..

[83] Hosseininejad Z., Moghadam S.D., Ebrahimi F., Abdollahi M., Zahedi M.J., Nazari M., Hayatbakhsh M., Adeli S., Sharififar F. (2011). In vitro screening of selected Iranian medicinal plants against *Helicobacter pylori*. Int. J. Green Pharm. (IJGP).

[84] Ali S.M., Khan A.A., Ahmed I., Musaddiq M., Ahmed K.S., Polasa H., Rao L.V., Habibullah C.M., Sechi L.A., Ahmed N. (2005). Antimicrobial activities of Eugenol and Cinnamaldehyde against the human gastric pathogen *Helicobacter pylori*. Ann. Clin. Microbiol. Antimicrob..

[85] Tabak M., Armon R., Neeman I. (1999). Cinnamon extracts’ inhibitory effect on *Helicobacter pylori*. J. Ethnopharmacol..

[86] Nabati F., Mojab F., Habibi-Rezaei M., Bagherzadeh K., Amanlou M., Yousefi B. (2012). Large scale screening of commonly used Iranian traditional medicinal plants against urease activity. Daru J. Pharm. Sci..

[87] Yeşilada E., Gürbüz I., Shibata H. (1999). Screening of Turkish anti-ulcerogenic folk remedies for anti-*Helicobacter pylori* activity. J. Ethnopharmacol..

[88] Ustün O., Ozçelik B., Akyön Y., Abbasoglu U., Yesilada E. (2006). Flavonoids with anti-Helicobacter pylori activity from *Cistus laurifolius* leaves. J. Ethnopharmacol..

[89] Bonamin F., Moraes T.M., Dos Santos R.C., Kushima H., Faria F.M., Silva M.A., Junior I.V., Nogueira L., Bauab T.M., Brito A.R.S., da Rocha L.R. (2014). The effect of a minor constituent of essential oil from *Citrus aurantium*: the role of β-myrcene in preventing peptic ulcer disease. Chem. Biol. Interact..

[90] Rozza A.L., de Mello Moraes T., Kushima H., Tanimoto A., Marques M.O.M., Bauab T.M., Hiruma-Lima C.A., Pellizzon C.H. (2011). Gastroprotective mechanisms of *Citrus lemon* (Rutaceae) essential oil and its majority compounds limonene and β-pinene: involvement of heat-shock protein-70, vasoactive intestinal peptide, glutathione, sulfhydryl compounds, nitric oxide and prostaglandin E2. Chem. Biol. Interact..

[91] Poovendran P., Kalaigandhi V., Poongunran E. (2011). Antimicrobial activity of the leaves of *Cocculus hirsutus* against gastric ulcer producing *Helicobacter pylori*. J. Pharm. Res..

[92] Njume C., Jide A.A., Ndip R.N. (2011). Aqueous and organic solvent-extracts of selected South African medicinal plants possess antimicrobial activity against drug-resistant strains of *Helicobacter pylori*: inhibitory and bactericidal potential. Int. J. Mol. Sci..

[93] Suleiman M.M., Tauheed M., Babandi J.S., Umar R., Sulaiman M.H., Shittu M., Isa H.I. (2013). An in vivo experimental trial to determine the efficacy of stem-bark extract of *Khaya senegalensis* A. Juss (Meliaceae) for treating gastric ulcer in rat. Int. J. Med. Aromat. Plants.

[94] Reyes‐Trejo B., Sánchez‐Mendoza M.E., Becerra‐García A.A., Cedillo‐Portugal E., Castillo‐Henkel C., Arrieta J. (2008). Bioassay‐guided isolation of an anti‐ulcer diterpenoid *from Croton reflexifolius*: role of nitric oxide, prostaglandins and sulfhydryls. J. Pharm. Pharmacol..

[95] Koga T., Kawada H., Utsui Y., Domon H., Ishii C., Yasuda H. (1996). In-vitro and in-vivo antibacterial activity of plaunotol, a cytoprotective antiulcer agent, against *Helicobacter pylori*. J. Antimicrob. Chemother..

[96] Nostro A., Cellini L., Bartolomeo S.D., Campli E.D., Grande R., Cannatelli M.A., Marzio L., Alonzo V. (2005). Antibacterial effect of plant extracts against *Helicobacter pylori*. Phytother. Res..

[97] Zaidi S.F.H., Yamada K., Kadowaki M., Usmanghani K., Sugiyama T. (2009). Bactericidal activity of medicinal plants, employed for the treatment of gastrointestinal ailments, against *Helicobacter pylori*. J. Ethnopharmacol..

[98] Ohno T., Kita M., Yamaoka Y., Imamura S., Yamamoto T., Mitsufuji S., Kodama T., Kashima K., Imanishi J. (2003). Antimicrobial activity of essential oils against *Helicobacter pylori*. Helicobacter.

[99] Jaguezeski A.M., Perin G., Crecencio R.B., Baldissera M.D., Stefanil L.M., da Silva A.S. (2018). Addition of curcumin in dairy sheep diet in the control of subclinical mastitis. Acta Sci. Vet..

[100] Escobedo-Hinojosa W.I., del Carpio J.D., Palacios-Espinosa J.F., Romero I. (2012). Contribution to the ethnopharmacological and anti-*Helicobacter pylori* knowledge of *Cyrtocarpa procera* Kunth (Anacardiaceae). J. Ethnopharmacol..

[101] Kushima H., Nishijima C.M., Rodrigues C.M., Rinaldo D., Sassá M.F., Bauab T.M., Di Stasi L.C., Carlos I.Z., Brito A.R.M.S., Vilegas W., Hiruma-Lima C.A. (2009). *Davilla elliptica* and *Davilla nitida*: gastroprotective, anti-inflammatory immunomodulatory and anti-*Helicobacter pylori* action. J. Ethnopharmacol..

[102] Bergonzelli G.E., Donnicola D., Porta N., Corthesy-Theulaz I.E. (2003). Essential oils as components of a diet-based approach to management of Helicobacter infection. Antimicrob. Agents Chemother..

[103] Uyub A.M., Nwachukwu I.N., Azlan A.A., Fariza S.S. (2010). http://hdl.handle.net/10125/21002.

[104] Ramadan M.A., Safwat N.A. (2009). Antihelicobacter activity of a flavonoid compound isolated from *Desmostachya bipinnata*. Aust. J. Basic Appl. Sci..

[105] Ibrahim N.H., Awaad A.S., Alnafisah R.A., Alqasoumi S.I., El-Meligy R.M., Mahmoud A.Z. (2018). In–vitro activity of *Desmostachya bipinnata* (L.) Stapf successive extracts against *Helicobacter pylori* clinical isolates. Saudi Pharm. J..

[106] Miguel G., Faleiro L., Cavaleiro C., Salgueiro L., Casanova J. (2008). Susceptibility of *Helicobacter pylori* to essential oil of *Dittrichia viscosa* subsp. revoluta. Phytother. Res..

[107] Adeniyi C.B.A., Lawal T.O., Mahady G.B. (2009). In vitro susceptibility of Helicobacter pylori to extracts of *Eucalyptus camaldulensis* and *Eucalyptus torelliana*. Pharm. Biol..

[108] Li Y., Xu C., Zhang Q., Liu J.Y., Tan R.X. (2005). In vitro anti-*Helicobacter pylori* action of 30 Chinese herbal medicines used to treat ulcer diseases. J. Ethnopharmacol..

[109] Sánchez-Mendoza M.E., Reyes-Trejo B., Sánchez-Gómez P., Rodríguez-Silverio J., Castillo-Henkel C., Cervantes-Cuevas H., Arrieta J. (2010). Bioassay-guided isolation of an anti-ulcer chromene from *Eupatorium aschenbornianum*: role of nitric oxide, prostaglandins and sulfydryls. Fitoterapia.

[110] Hamasaki N., Ishii E., Tominaga K., Tezuka Y., Nagaoka T., Kadota S., Kuroki T., Yano I. (2000). Highly selective antibacterial activity of novel alkyl quinolone alkaloids from a Chinese herbal medicine, Gosyuyu (Wu-Chu-Yu), against *Helicobacter pylori* in vitro. Microbiol. Immunol..

[111] Basile A., Conte B., Rigano D., Senatore F., Sorbo S. (2010). Antibacterial and antifungal properties of acetonic extract of Feijoa sellowiana fruits and its effect on *Helicobacter pylori* growth. J. Med. Food.

[112] Basile A., Sorbo S., Spadaro V., Bruno M., Maggio A., Faraone N., Rosselli S. (2009). Antimicrobial and antioxidant activities of coumarins from the roots of *Ferulago campestris* (Apiaceae). Molecules.

[113] Rosselli S., Maggio A.M., Faraone N., Spadaro V., Morris-Natschke S.L., Bastow K.F., Lee K.H., Bruno M. (2009). The cytotoxic properties of natural coumarins isolated from roots of *Ferulago campestris* (Apiaceae) and of synthetic ester derivatives of aegelinol. Nat. Prod. Commun..

[114] Jadhav S.G., Meshram R.J., Gond D.S., Gacche R.N. (2013). Inhibition of growth of *Helicobacter pylori* and its urease by coumarin derivatives: molecular docking analysis. J. Pharm. Res..

[115] Kawase M., Tanaka T., Sohara Y., Tani S., Sakagami H., Hauer H., Chatterjee S.S. (2003). Structural requirements of hydroxylated coumarins for in vitro anti-*Helicobacter pylori* activity. In Vivo.

[116] Niero R., Dal Molin M.M., Silva S., Damian N.S., Maia L.O., Delle Monache F., Cechinel Filho V., de Andrade S.F. (2012). Gastroprotective effects of extracts and guttiferone A isolated from *Garcinia achachairu* Rusby (Clusiaceae) against experimentally induced gastric lesions in mice. Naunyn Schmiedebergs Arch. Pharmacol..

[117] Zhang X.Q., Gu H.M., Li X.Z., Xu Z.N., Chen Y.S., Li Y. (2013). Anti-*Helicobacter pylori* compounds from the ethanol extracts of Geranium wilfordii. J. Ethnopharmacol..

[118] Shahani S., Monsef-Esfahani H.R., Saeidnia S., Saniee P., Siavoshi F., Foroumadi A., Samadi N., Gohari A.R. (2012). Anti-*Helicobacter pylori* activity of the methanolic extract of *Geum iranicum* and its main compounds. Z. Naturforschung C.

[119] Fukai T., Marumo A., Kaitou K., Kanda T., Terada S., Nomura T. (2002). Anti-*Helicobacter pylori* flavonoids from licorice extract. Life Sci..

[120] Aly A.M., Al-Alousi L., Salem H.A. (2005). Licorice: a possible anti-inflammatory and anti-ulcer drug. AAPS PharmSciTech.

[121] de Mello Moraes T., Rodrigues C.M., Kushima H., Bauab T.M., Villegas W., Pellizzon C.H., Brito A.R.M.S., Hiruma-Lima C.A. (2008). Hancornia speciosa: indications of gastroprotective, healing and anti-*Helicobacter pylori* actions. J. Ethnopharmacol..

[122] Shang X., Tan Q., Liu R., Yu K., Li P., Zhao G.P. (2013). In vitro anti-*Helicobacter pylori* effects of medicinal mushroom extracts, with special emphasis on the Lion’s Mane mushroom, *Hericium erinaceus* (higher Basidiomycetes). Int. J. Med. Mushrooms.

[123] Mahady G.B., Pendland S.L., Stoia A., Chadwick L.R. (2003). In vitro susceptibility of Helicobacter pylori to isoquinoline alkaloids from *Sanguinaria canadensis* and *Hydrastis canadensis*. Phytother. Res..

[124] Mohtar M., Johari S.A., Li A.R., Isa M.M., Mustafa S., Ali A.M., Basri D.F. (2009). Inhibitory and resistance-modifying potential of plant-based alkaloids against methicillin-resistant *Staphylococcus aureus* (MRSA). Curr. Microbiol..

[125] Markham P.N., Westhaus E., Klyachko K., Johnson M.E., Neyfakh A.A. (1999). Multiple novel inhibitors of the NorA multidrug transporter of *Staphylococcus aureus*. Antimicrob. Agents Chemother..

[126] Rao K.N., Venkatachalam S.R. (2000). Inhibition of dihydrofolate reductase and cell growth activity by the phenanthroindolizidine alkaloids pergularinine and tylophorinidine: the in vitro cytotoxicity of these plant alkaloids and their potential as antimicrobial and anticancer agents. Toxicol. Vitro.

[127] Vera-Arzave C., Antonio L.C., Arrieta J., Cruz-Hernández G., Velázquez-Méndez A.M., Reyes-Ramírez A., Sánchez-Mendoza M.E. (2012). Gastroprotection of suaveolol, isolated from *Hyptis suaveolens*, against ethanol-induced gastric lesions in Wistar rats: role of prostaglandins, nitric oxide and sulfhydryls. Molecules.

[128] Lu M.C., Chiu H.F., Lin C.P., Shen Y.C., Venkatakrishnan K., Wang C.K. (2018). Anti-*Helicobacter pylori* effect of various extracts of ixeris chinensis on inflammatory markers in human gastric epithelial AGS cells. J. Herb. Med..

[129] Pertino M., Schmeda-Hirschmann G., Rodríguez J.A., Theoduloz C. (2007). Gastroprotective effect and cytotoxicity of terpenes from the Paraguayan crude drug “yagua rova” (Jatropha isabelli). J. Ethnopharmacol..

[130] Hajimahmoodi M., Shams-Ardakani M., Saniee P., Siavoshi F., Mehrabani M., Hosseinzadeh H., Foroumadi P., Safavi M., Khanavi M., Akbarzadeh T., Shafiee A. (2011). In vitro antibacterial activity of some Iranian medicinal plant extracts against *Helicobacter pylori*. Nat. Prod. Res..

[131] Stege P.W., Davicino R.C., Vega A.E., Casali Y.A., Correa S., Micalizzi B. (2006). Antimicrobial activity of aqueous extracts of *Larrea divaricata* Cav (jarilla) against *Helicobacter pylori*. Phytomedicine.

[132] Bae E.A., Han M.J., Kim N.J., Kim D.H. (1998). Anti-*Helicobacter pylori* activity of hearbal medicines. Biol. Pharm. Bull..

[133] Neelima N., Sudhakar M., Patil M.B., Lakshmi B.V.S. (2012). Anti-ulcer activity and HPTLC analysis of *Mangifera indica* L. leaves. Int. J. Pharm. Phytopharm. Res..

[134] Carvalho A.C.S., Guedes M.M., de Souza A.L., Trevisan M.T., Lima A.F., Santos F.A., Rao V.S. (2007). Gastroprotective effect of mangiferin, a xanthonoid from *Mangifera indica*, against gastric injury induced by ethanol and indomethacin in rodents. Planta Med..

[135] Abdulla M.A., Ali H.M., Ahmed K.A.A., Noor S.M., Ismail S. (2009).

[136] Ahmad A., Gupta G., Afzal M., Kazmi I., Anwar F. (2013). Antiulcer and antioxidant activities of a new steroid from *Morus alba*. Life Sci..

[137] Sidahmed H.M.A., Azizan A.H.S., Mohan S., Abdulla M.A., Abdelwahab S.I., Taha M.M.E., Hadi A.H.A., Ketuly K.A., Hashim N.M., Loke M.F., Vadivelu J. (2013). Gastroprotective effect of desmosdumotin C isolated from *Mitrella kentii* against ethanol-induced gastric mucosal hemorrhage in rats: possible involvement of glutathione, heat-shock protein-70, sulfhydryl compounds, nitric oxide, and anti-*Helicobacter pylori* activity. BMC Complement. Altern. Med..

[138] Lewis D.A., Shaw G.P. (2001). A natural flavonoid and synthetic analogues protect the gastric mucosa from aspirin-induced erosions. J. Nutr. Biochem..

[139] Jain D.L., Baheti A.M., Parakh S.R., Ingale S.P., Ingale P.L. (2007). PHCOG MAG.: research article study of antacid and diuretic activity of ash and extracts of *Musa sapientum* L. fruit peel. Phcog. Mag..

[140] Bhamarapravati S., Pendland S.L., Mahady G.B. (2003). Extracts of spice and food plants from Thai traditional medicine inhibit the growth of the human carcinogen *Helicobacter pylori*. In Vivo (Athens, Greece).

[141] Ohsaki A., Takashima J., Chiba N., Kawamura M. (1999). Microanalysis of a selective potent anti-*Helicobacter pylori* compound in a Brazilian medicinal plant, *Myroxylon peruiferum* and the activity of analogues. Bioorg. Med. Chem. Lett..

[142] Deriu A., Branca G., Molicotti P., Pintore G., Chessa M., Tirillini B., Paglietti B., Mura A., Sechi L.A., Fadda G., Zanetti S. (2007). In vitro activity of essential oil of *Myrtus communis* L. against *Helicobacter pylori*. Int. J. Antimicrob. Agents.

[143] Sudjana A.N., D’Orazio C., Ryan V., Rasool N., Ng J., Islam N., Riley T.V., Hammer K.A. (2009). Antimicrobial activity of commercial *Olea europaea* (olive) leaf extract. Int. J. Antimicrob. Agents.

[144] Singh S., Majumdar D.K. (1999). Evaluation of the gastric antiulcer activity of fixed oil of *Ocimum sanctum* (Holy Basil). J. Ethnopharmacol..

[145] Sumbul S., Ahmad M.A., Mohd A., Mohd A. (2011). Role of phenolic compounds in peptic ulcer: an overview. J. Pharm. Bioallied Sci..

[146] Ares J.J., Outt P.E., Randall J.L., Johnston J.N., Murray P.D., O’Brien L.M., Weisshaar P.S., Ems B.L. (1996). Synthesis and biological evaluation of flavonoids and related compounds as gastroprotective agents. Bioorg. Med. Chem. Lett..

[147] Afroz S., Yagi A., Fujikawa K., Rahman M.M., Morito K., Fukuta T., Watanabe S., Kiyokage E., Toida K., Shimizu T., Ishida T. (2018). Lysophosphatidic acid in medicinal herbs enhances prostaglandin E2 and protects against indomethacin-induced gastric cell damage in vivo and in vitro. Prostaglandins Other Lipid Mediat..

[148] Sun X.B., Matsumolo T., Yamada H. (1992). Purification of an anti-ulcer polysaccharide from the leaves of *Panax ginseng*. Planta Med..

[149] Yamada H. (1994). Pectic polysaccharides from Chinese herbs: structure and biological activity. Carbohydr. Polym..

[150] Cowan M.M. (1999). Plant products as antimicrobial agents. Clin. Microbiol. Rev..

[151] Rojas-Martínez R., Arrieta J., Cruz-Antonio L., Arrieta-Baez D., Velázquez-Méndez A., Sánchez-Mendoza M. (2013). Dillapiole, isolated from *Peperomia pellucida*, shows gastroprotector activity against ethanol-induced gastric lesions in Wistar rats. Molecules.

[152] Ranilla L.G., Apostolidis E., Shetty K. (2012). Antimicrobial activity of an Amazon medicinal plant (Chancapiedra) (*Phyllanthus niruri* L.) against *Helicobacter pylori* and lactic acid bacteria. Phytother. Res..

[153] Wang Y., Wang S.L., Zhang J.Y., Song X.N., Zhang Z.Y., Li J.F., Li S. (2018). Anti-ulcer and anti-*Helicobacter pylori* potentials of the ethyl acetate fraction of *Physalis alkekengi* L. var. franchetii (Solanaceae) in rodent. J. Ethnopharmacol..

[154] Quílez A., Berenguer B., Gilardoni G., Souccar C., De Mendonça S., Oliveira L.F.S., Martín-Calero M.J., Vidari G. (2010). Anti-secretory, anti-inflammatory and anti-Helicobacter pylori activities of several fractions isolated from *Piper carpunya* Ruiz & Pav. J. Ethnopharmacol..

[155] Rüegg T., Calderón A.I., Queiroz E.F., Solis P.N., Marston A., Rivas F., Ortega-Barría E., Hostettmann K., Gupta M.P. (2006). 3-Farnesyl-2-hydroxybenzoic acid is a new anti-*Helicobacter pylori* compound from *Piper multiplinervium*. J. Ethnopharmacol..

[156] Al-Said M.S., Ageel A.M., Parmar N.S., Tariq M. (1986). Evaluation of mastic, a crude drug obtained from *Pistacia lentiscus* for gastric and duodenal anti-ulcer activity. J. Ethnopharmacol..

[157] Marone P., Bono L., Leone E., Bona S., Carretto E., Perversi L. (2001). Bactericidal activity of *Pistacia lentiscus* mastic gum against *Helicobacter pylori*. J. Chemother..

[158] Dabos K.J., Sfika E., Vlatta L.J., Giannikopoulos G. (2010). The effect of mastic gum on *Helicobacter pylori*: a randomized pilot study. Phytomedicine.

[159] Paraschos S., Magiatis P., Mitakou S., Petraki K., Kalliaropoulos A., Maragkoudakis P., Mentis A., Sgouras D., Skaltsounis A.L. (2007). In vitro and in vivo activities of Chios mastic gum extracts and constituents against *Helicobacter pylori*. Antimicrob. Agents Chemother..

[160] de Araújo Rodrigues P., de Morais S.M., de Souza C.M., Silva A.R.A., de Andrade G.M., Silva M.G.V., Albuquerque R.L., Rao V.S., Santos F.A. (2010). Gastroprotective effect of barbatusin and 3-beta-hydroxy-3-deoxibarbatusin, quinonoid diterpenes isolated from *Plectranthus grandis*, in ethanol-induced gastric lesions in mice. J. Ethnopharmacol..

[161] Wang Y.C., Huang T.L. (2005). High-performance liquid chromatography for quantification of plumbagin, an anti-*Helicobacter pylori* compound of *Plumbago zeylanica* L. J. Chromatogr. A.

[162] Klein L.C., Gandolfi R.B., Santin J.R., Lemos M., Cechinel Filho V., de Andrade S.F. (2010). Antiulcerogenic activity of extract, fractions, and some compounds obtained from *Polygala cyparissias* St. Hillaire & Moquin (Polygalaceae). Naunyn Schmiedeberg’s Arch. Pharmacol..

[163] Hashimoto T., Aga H., Chaen H., Fukuda S., Kurimoto M. (1999). Isolation and identification of anti-*Helicobacter pylori* compounds from *Polygonum tinctorium* Lour. Nat. Med.= 生薬學雜誌.

[164] Tomczyk M., Leszczyńska K., Jakoniuk P. (2008). Antimicrobial activity of *Potentilla* species. Fitoterapia.

[165] Bisignano C., Filocamo A., La Camera E., Zummo S., Fera M.T., Mandalari G. (2013). Antibacterial activities of almond skins on cagA-positive and-negative clinical isolates of *Helicobacter pylori*. BMC Microbiol..

[166] Yin S., Fan C.Q., Dong L., Yue J.M. (2006). Psoracorylifols A–E, five novel compounds with activity against *Helicobacter pylori* from seeds of *Psoralea corylifolia*. Tetrahedron.

[167] De Leo M., De Tommasi N., Sanogo R., D’Angelo V., Germanò M.P., Bisignano G., Braca A. (2006). Triterpenoid saponins from *Pteleopsis suberosa* stem bark. Phytochemistry.

[168] Voravuthikunchai S.P., Limsuwan S., Mitchell H. (2006). Effects of *Punica granatum* pericarps and *Quercus infectoria* nutgalls on cell surface hydrophobicity and cell survival of *Helicobacter pylori*. J. Health Sci..

[169] Moghaddam M.N. (2011). In vitro inhibition of *Helicobacter pylori* by some spices and medicinal plants used in Iran. Glob. J. Pharmacol..

[170] Mazzolin L.P., Nasser A.L.M., Moraes T.M., Santos R.C., Nishijima C.M., Santos F.V., Varanda E.A., Bauab T.M., da Rocha L.R.M., Di Stasi L.C., Vilegas W. (2010). *Qualea parviflora* Mart.: an integrative study to validate the gastroprotective, antidiarrheal, antihemorragic and mutagenic action. J. Ethnopharmacol..

[171] Kadota S., Basnet P., Ishii E., Tamura T., Namba T. (1997). Antibacterial activity of trichorabdal A from *Rabdosia trichocarpa* against *Helicobacter pylori*. Zent. Bakteriol..

[172] Chung J.G., Tsou M.F., Wang H.H., Lo H.H., Hsieh S.E., Yen Y.S., Wu L.T., Chang S.H., Ho C.C., Hung C.F. (1998). Rhein affects arylamine N‐acetyltransferase activity in *Helicobacter pylori* from peptic ulcer patients. J. Appl.Toxicol..

[173] Berté P.E., da Silva Lopes J., Comandulli N.G., Rangel D.W., Delle Monache F., Cechinel Filho V., Niero R., de Andrade S.F. (2014). Evaluation of the gastroprotective activity of the extracts, fractions, and pure compounds obtained from aerial parts of Rubus imperialis in different experimental models. Naunyn Schmiedebergs Arch. Pharmacol..

[174] Atapour M., Zahedi M.J., Mehrabani M., Safavi M., Keyvanfard V., Foroughi A., Siavoshi F., Foroumadi A. (2009). In vitro susceptibility of the Gram-negative bacterium *Helicobacter pylori* to extracts of Iranian medicinal plants. Pharm. Biol..

[175] Gharashi A. (2008).

[176] Ochi T., Shibata H., Higuti T., Kodama K.H., Kusumi T., Takaishi Y. (2005). Anti-*Helicobacter pylori* compounds from *Santalum album*. J. Nat. Prod..

[177] La Casa C., Villegas I., De La Lastra C.A., Motilva V., Calero M.M. (2000). Evidence for protective and antioxidant properties of rutin, a natural flavone, against ethanol induced gastric lesions. J. Ethnopharmacol..

[178] Njume C., Afolayan A.J., Green E., Ndip R.N. (2011). Volatile compounds in the stem bark of *Sclerocarya birrea* (Anacardiaceae) possess antimicrobial activity against drug-resistant strains of *Helicobacter pylori*. Int. J. Antimicrob. Agents.

[179] Júnior G.M.V., da Rocha C.Q., de Souza Rodrigues T., Hiruma-Lima C.A., Vilegas W. (2015). New steroidal saponins and antiulcer activity from *Solanum paniculatum* L. Food Chem..

[180] Areche C., Schmeda‐Hirschmann G., Theoduloz C., Rodríguez J.A. (2009). Gastroprotective effect and cytotoxicity of abietane diterpenes from the Chilean Lamiaceae sphacele chamaedryoides (Balbis) Briq. J. Pharm. Pharmacol..

[181] Khanavi M., Safavi M., Siavoi F., Fallah Tafti A., Hajimahmoodi M., Hadjiakhoondi A., Rezazadeh S., Foroumadi A. (2008). Evaluation of anti-*Helicobacter pylori* activity of methanol extracts of some species of Stachys and Melia. J. Med. Plants.

[182] Bonamin F., Moraes T.M., Kushima H., Silva M.A., Rozza A.L., Pellizzon C.H., Bauab T.M., Rocha L.R.M., Vilegas W., Hiruma-Lima C.A. (2011). Can a *Strychnos* species be used as antiulcer agent? Ulcer healing action from alkaloid fraction of *Strychnos pseudoquina* St. Hil.(Loganiaceae). J. Ethnopharmacol..

[183] Magaji R.A., Okasha M.A.M., Abubakar M.S., Fatihu M.Y. (2007). Anti-ulcerogenic and anti-secretory activity of the n-butanol portion of *Syzygiumaromaticum* in rat. Nig. J. Pharm. Sci..

[184] Babu T.H., Manjulatha K., Kumar G.S., Hymavathi A., Tiwari A.K., Purohit M., Rao J.M., Babu K.S. (2010). Gastroprotective flavonoid constituents from *Oroxylum indicum* Vent. Bioorg. Med. Chem. Lett..

[185] Park B.S., Lee H.K., Lee S.E., Piao X.L., Takeoka G.R., Wong R.Y., Ahn Y.J., Kim J.H. (2006). Antibacterial activity of *Tabebuia impetiginosa* Martius ex DC (Taheebo) against *Helicobacter pylori*. J. Ethnopharmacol..

[186] Chatterjee A., Khatua S., Chatterjee S., Mukherjee S., Mukherjee A., Paloi S., Acharya K., Bandyopadhyay S.K. (2013). Polysaccharide-rich fraction of *Termitomyces eurhizus* accelerate healing of indomethacin induced gastric ulcer in mice. Glycoconj. J..

[187] Fabry W., Okemo P., Ansorg R. (1996). Activity of East African medicinal plants against *Helicobacter pylori*. Chemotherapy.

[188] Malekzadeh F., Ehsanifar H., Shahamat M., Levin M., Colwell R.R. (2001). Antibacterial activity of black myrobalan (*Terminalia chebula* Retz) against *Helicobacter pylori*. Int. J. Antimicrob. Agents.

[189] Mishra V., Agrawal M., Onasanwo S.A., Madhur G., Rastogi P., Pandey H.P., Palit G., Narender T. (2013). Anti-secretory and cyto-protective effects of chebulinic acid isolated from the fruits of *Terminalia chebula* on gastric ulcers. Phytomedicine.

[190] Bag A., Bhattacharyya S.K., Chattopadhyay R.R. (2013). The development of *Terminalia chebula* Retz. (Combretaceae) in clinical research. Asian Pac. J. Trop. Biomed..

[191] Esmaeili D., Mobarez A.M., Tohidpour A. (2012). Anti-*Helicobacter pylori* activities of shoya powder and essential oils of thymus vulgaris and eucalyptus globulus. Open Microbiol. J..

[192] Sánchez-Mendoza M.E., Reyes-Ramírez A., Cruz Antonio L., Martínez Jiménez L., Rodríguez-Silverio J., Arrieta J. (2011). Bioassay-guided isolation of an anti-ulcer compound, tagitinin C, from *Tithonia diversifolia*: role of nitric oxide, prostaglandins and sulfhydryls. Molecules.

[193] Nariman F., Eftekhar F., Habibi Z., Falsafi T. (2004). Anti-*Helicobacter pylori* activities of six Iranian plants. Helicobacter.

[194] Burger O., Ofek I., Tabak M., Weiss E.I., Sharon N., Neeman I. (2000). A high molecular mass constituent of cranberry juice inhibits *Helicobacter pylori* adhesion to human gastric mucus. FEMS Immunol. Med. Microbiol..

[195] Shmuely H., Burger O., Neeman I., Yahav J., Samra Z., Niv Y., Sharon N., Weiss E., Athamna A., Tabak M., Ofek I. (2004). Susceptibility of *Helicobacter pylori* isolates to the antiadhesion activity of a high-molecular-weight constituent of cranberry. Diagn. Microbiol. Infect. Dis..

[196] Ghannadi A., Sajjadi S.E., Abedi D., Yousefi J., Daraei-Ardekani R. (2004). The in vitro activity of seven Iranian plants of the Lamiaceae family against *Helicobacter pylori*. Niger. J. Nat. Prod. Med..

[197] Backon J. (1986). Ginger: inhibition of thromboxane synthetase and stimulation of prostacyclin: relevance for medicine and psychiatry. Med. Hypotheses.

[198] Johji Y., Michihiko M., Rong H.Q., Hisashi M., Hajime F. (1988). The anti-ulcer effect in rats of ginger constituents. J. Ethnopharmacol..

[199] Al-Yahya M.A., Rafatullah S., Mossa J.S., Ageel A.M., Parmar N.S., Tariq M. (1989). Gastroprotective activity of ginger zingiber officinale rosc., in albino rats. Am. J. Chin. Med..

[200] Yoshikawa M., Hatakeyama S., Taniguchi K., Matuda H., Yamahara J. (1992). 6-gingesulfonic acid, a new anti-ulcer principle, and gingerglycolipids A, B and C, three new monoacyldigalactosylglycerols, from *Zingiberis rhizoma* originating in Taiwan. Chem. Pharm. Bull..

[201] Yoshikawa M., Yamaguchi S., Kunimi K., Matsuda H., Okuno Y., Yamahara J., Murakami N. (1994). Stomachic principles in ginger. III. An anti-ulcer principle, 6-gingesulfonic acid, and three monoacyldigalactosylglycerols, gingerglycolipids A, B, and C, from *Zingiberis Rhizoma* originating in Taiwan. Chem. Pharm. Bull..

[202] Banerjee S., Mullick H.I., Banerjee J., Ghosh A. (2011). Zingiber officinale: ‘a natural gold’. Int. J. Pharm. Biol.-Sci..

